# Supplementary material for: Neonatal Gene Therapy for Hemophilia B by a Novel Adenovirus Vector Showing Reduced Leaky Expression of Viral Genes
Source: Mol Ther Methods Clin Dev. 2017 Jul 8;6:183–93. doi: 10.1016/j.omtm.2017.07.001 (PMC5552065; doi:10.1016/j.omtm.2017.07.001)
Supplement: Document S1. Supplemental Materials and Methods, Figures S1–S10, and Table S1 [file mmc1.pdf]

OMTM, Volume *6*

Supplemental Information

Neonatal Gene Therapy for Hemophilia B by a Novel Adenovirus Vector Showing Reduced Leaky Expression of Viral Genes

Shunsuke Iizuka, Fuminori Sakurai, Masashi Tachibana, Kazuo Ohashi, and Hiroyuki Mizuguchi

Supplementary materials and methods

Analysis of serum hepatotoxicity marker

Blood samples were collected and centrifuged (4°C, 7,000 g, 15 min) to obtain the serum. The serum aspartateaminotransferase (AST) levels were determined using a transaminase-CII kit (Wako Pure Chemical).

Real-time RT PCR analysis

Total RNA was extracted from the liver and spleen using ISOGEN (Wako Pure Chemical, Osaka, Japan). mRNA levels of cytokines, including interleukin-(IL-) 6, IL-12, interferon (IFN) -γ, and glyceroaldehyde-3-phosphatedehydrogenase (GAPDH), and albumin were determined by real-time RT-PCR using THUNDERBIRD SYBR qPCR Mix (Toyobo, Osaka, Japan), as previously described [1].

Detection of anti-Ad antibody

Ad-AHAmSEAP and Ad-E4-122aT-AHAmSEAP were administered to neonatal mice *via* the retro-orbital sinus. On day 42 after the 1st injection, Ad vectors were sequentially administered *via* the tail vein. Serum samples were collected as described above on day 56 following neonatal administration. Anti-Ad antibody titers were determined by ELISA as previously described [2]. 96-well immune-plate (Thermo Fisher Scientific, Waltham, MA) was pre-coated with $5x10^{8}$ vp of Ad-null/well. Serum samples were diluted to 1:2,560.

Anlysis of Hexon specific CTL responses

Ad-AHAmSEAP and Ad-E4-122aT-AHAmSEAP were administered to neonatal mice *via* the retro-orbital sinus. On day 42 after the 1st injection, Ad vectors were sequentially administered *via* the tail vein. Splenocytes were harvested on day 56 following 1st injection. Ad hexon specific CTLs in the splenocytes were examined using a Cytofix/CytoPerm Plus kit (BD Biosciences, San Diego, CA). Briefly, $2x10^{6}$ splenocytes were cultured with Ad hexon peptide pool (Milteny Biotec, Bergisch Gladbach, Germany), co-stimulation antibody (anti-mouse CD28 and CD49d; eBioscience, San Diego, CA) and GolgiStop (BD Biosciences) in RPMI-1640 (Sigma-Aldrich, St. Louis, MO). Six hours after incubation, splenocytes were stained for viability using Live/ Dead Fixable Dead Cell Stain Kits (Invitrogen, Carlsbad, CA) for 30 min at room temperature. Following incubation and washing with PBS, splenocytes were stained with phycoerythrin (PE)-conjugated anti-mouse CD3ε antibody (eBioscience) and allophycocyanin (APC)-Cy7-conjugated anti-mouse CD8 antibody (Biolegend, San Diego, CA) for 30 min at 4°C. After incubation and washing with PBS, splenocytes were incubated in Cytofix/Cytoperm solution for 30 min at 4°C for permeabilization and stained with PE-Cy7-conjugated anti-mouse interferon (IFN)-γ antibody (eBioscience). Data were analyzed using FlowJo software (TreeStar, Ashland, OR).

Supplementary references

[1] S. Iizuka, F. Sakurai, K. Shimizu, K. Ohashi, S.-I. Nakamura, M. Tachibana, H. Mizuguchi, Evaluation of transduction properties of an adenovirus vector in neonatal mice, Biomed Res. Int. 2015 (2015). doi:10.1155/2015/685374.

[2] K. Shimizu, F. Sakurai, K. Tomita, Y. Nagamoto, S. Nakamura, K. Katayama, M. Tachibana, K. Kawabata, H. Mizuguchi, Suppression of leaky expression of adenovirus genes by insertion of microRNA-targeted sequences in the replication-incompetent adenovirus vector genome, Mol. Ther. — Methods Clin. Dev. 1 (2014) 14035. doi:10.1038/mtm.2014.35.

Figure S1

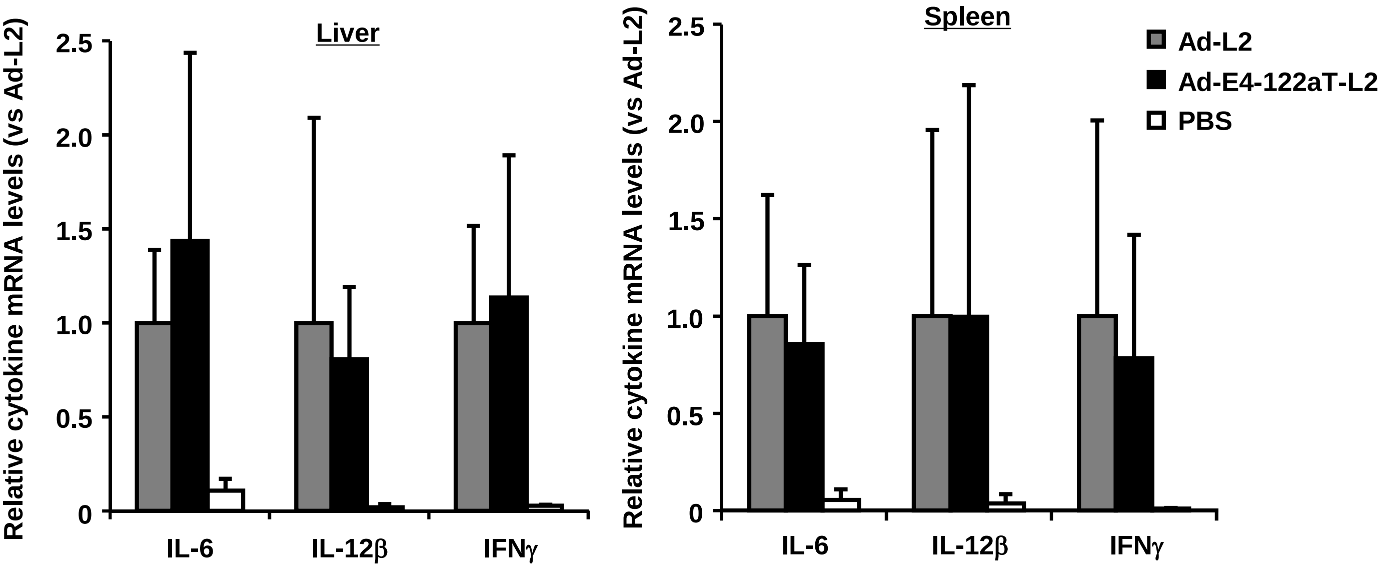

Figure S1. Innate immune responses in the neonatal liver and spleen following systemic administration of Ad vectors. Neonatal mice were administered Ad vectors at a dose of 5.9×10^{11} IFU/kg *via* the retro-orbital sinus. The liver and spleen were collected 6 h after Ad vector administration to recover total RNA. All values were normalized to 1 by the data from the Ad-L2-injected group. Results are shown as the averages ± S.D. (n=5-6).

Figure S2

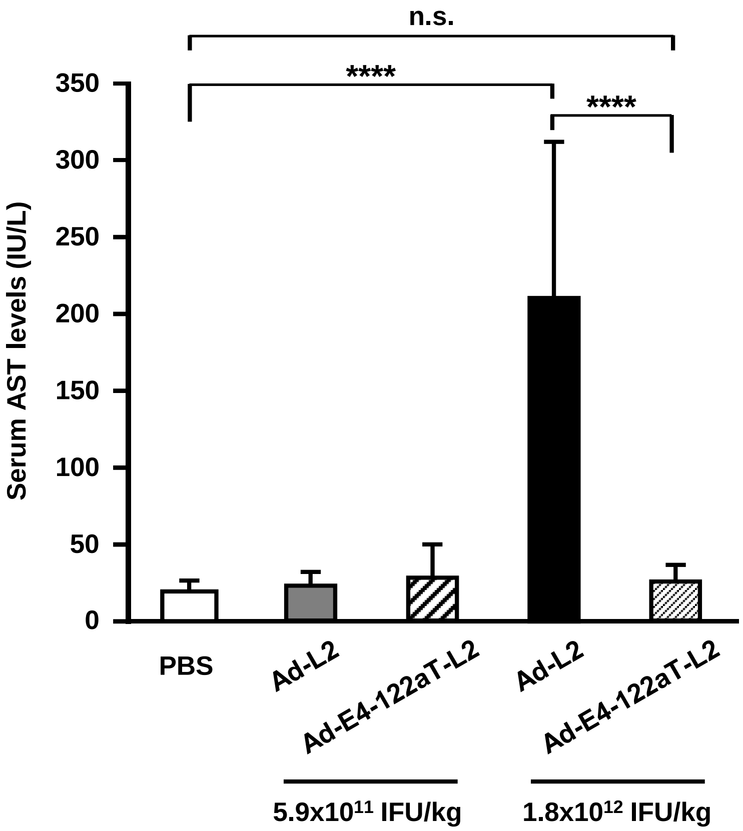

Figure S2. Serum AST levels following Ad vector administration in neonatal mice. Neonatal mice were administered Ad vectors at a dose of 5.9x10^{11} IFU/kg or 1.8x10^{12} IFU/kg *via* the retro-orbital sinus. On day 2 following Ad vector administration, serum AST levels were measured. Results are shown as the averages ± S.D. (n=7-8). AST, aspartate aminotransferase; n.s., not significant. ****$p < 0.0001$.

Figure S3

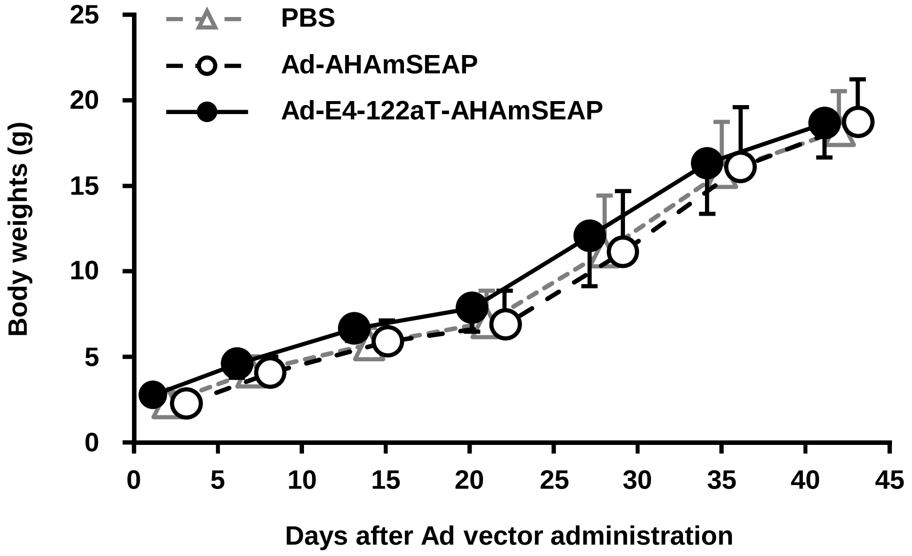

Figure S3. Mouse body weights following Ad vector administration in neonatal mice. Neonatal mice were administered Ad vectors at a dose of $5.9x10^{11}$ IFU/kg *via* the retro-orbital sinus. Body weights were measured at the indicated time points. Results are shown as the averages ± S.D. (n=7-8).

Figure S4

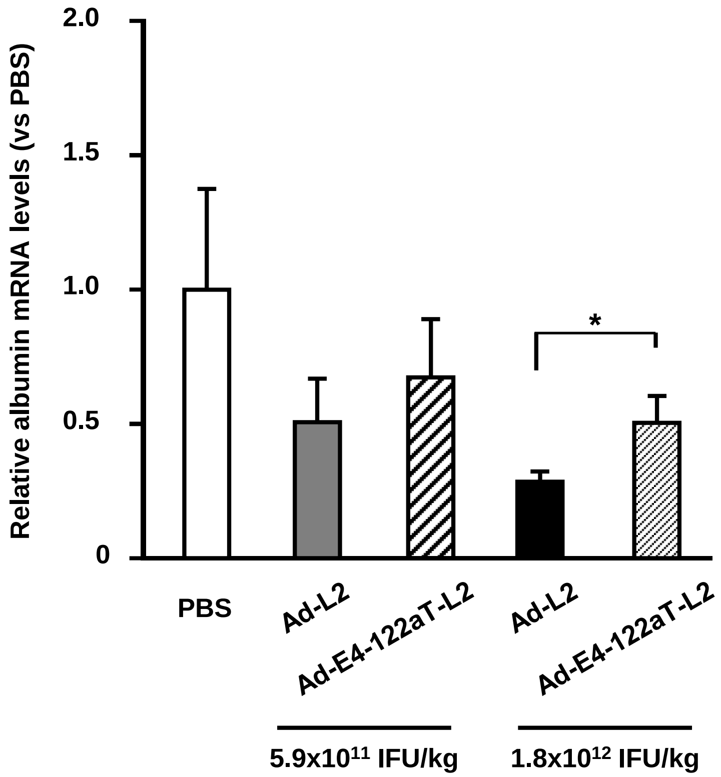

Figure S4. Endogenous albumin expression levels in the neonatal liver following systemic administration of Ad vectors. Neonatal mice were administered Ad vectors at a dose of 5.9×10^{11} IFU/kg or 1.8×10^{12} IFU/kg *via* the retro-orbital sinus. The liver was harvested to recover total RNA on day 2 following Ad vector administration. Endogenous albumin expression levels were measured by real-time RT-PCR. All values were normalized to 1 by the data from the PBS-injected group. Results are shown as the averages ± S.D. (n=6). n.s., not significant. *$p < 0.05$. ** $p < 0.01$.

Table S1

Table S1. Viability of neonatal mice following systemic administration of high doses of Ad vectors.

Injection titer	*Ad vector*	*Number of injected mice*	*Number of dead mice*	*Viability*
$1.8x10^{12}$ IFU/kg	Ad-L2	9	2	77.8%
	Ad-E4-122aT-L2	9	0	100%
$2.9x10^{12}$ IFU/kg	Ad-L2	6	3	50%
	Ad-E4-122aT-L2	6	0	100%

Neonatal mice were administered Ad vectors at a dose of $1.8x10^{12}$ IFU/kg or $2.9x10^{12}$ IFU/kg *via* the retro-orbital sinus. Two days after transduction, viabilities of mice were examined.

Figure S5

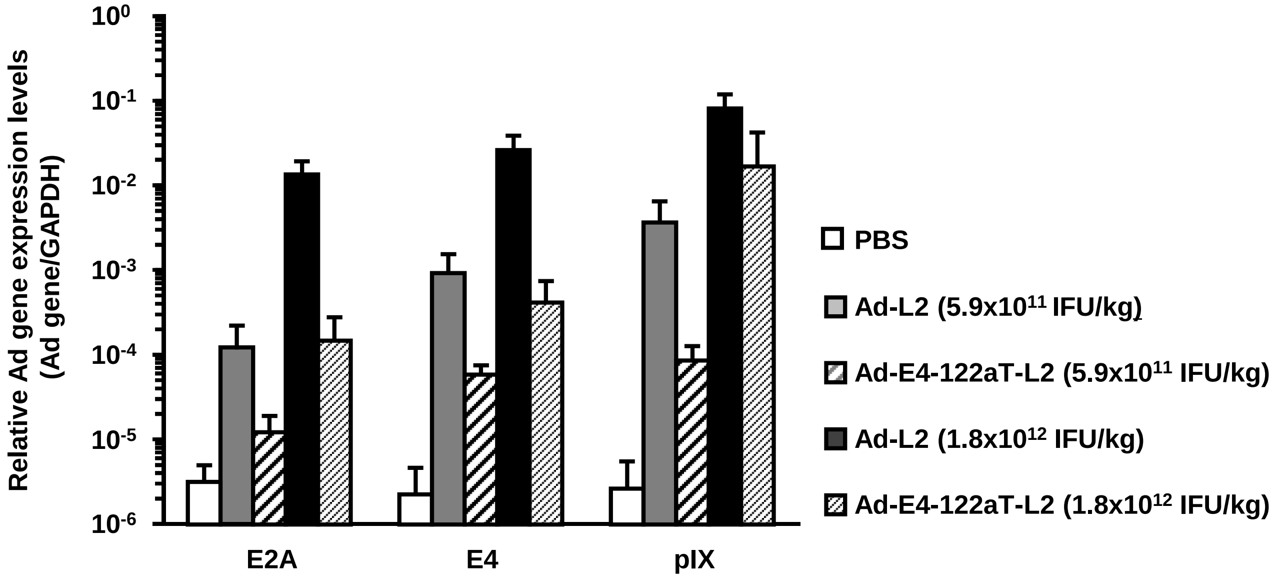

Figure S5. Leaky expression of Ad genes in the neonatal mouse liver following systemic administration of Ad vectors. Neonatal mice were administered Ad vectors at a dose of $5.9x10^{11}$ IFU/kg or $1.8x10^{12}$ IFU/kg *via* the retro-orbital sinus. Two days after transduction, the Ad gene expression levels in the liver were determined by real-time RT-PCR. Results are shown as the averages ± S.D. (n=5-6).

Figure S6

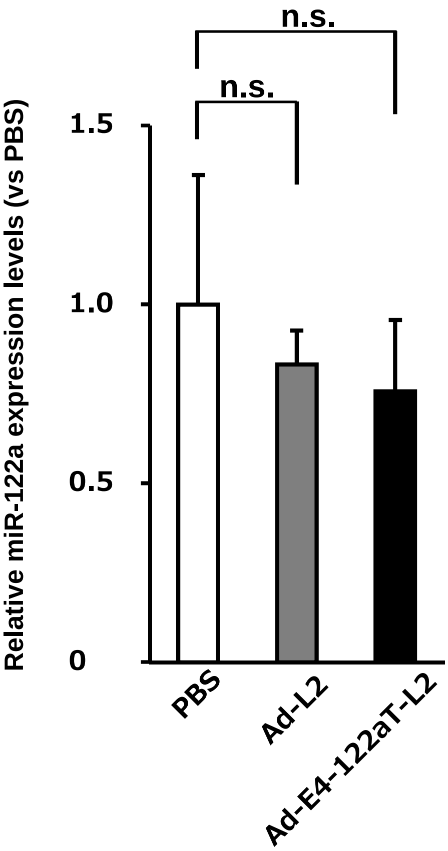

Figure S6. miR-122a expression levels in the neonatal liver following systemic administration of Ad vectors. Neonatal mice were administered Ad vectors at a dose of 5.9×10^{11} IFU/kg *via* the retro-orbital sinus. The liver was harvested to recover total RNA on day 2 following Ad vector administration. miR-122a expression levels were measured by real-time RT-PCR. All values were normalized to 1 by the data from the PBS-injected group. Results are shown as the averages ± S.D. (n=6). n.s., not significant.

Figure S7

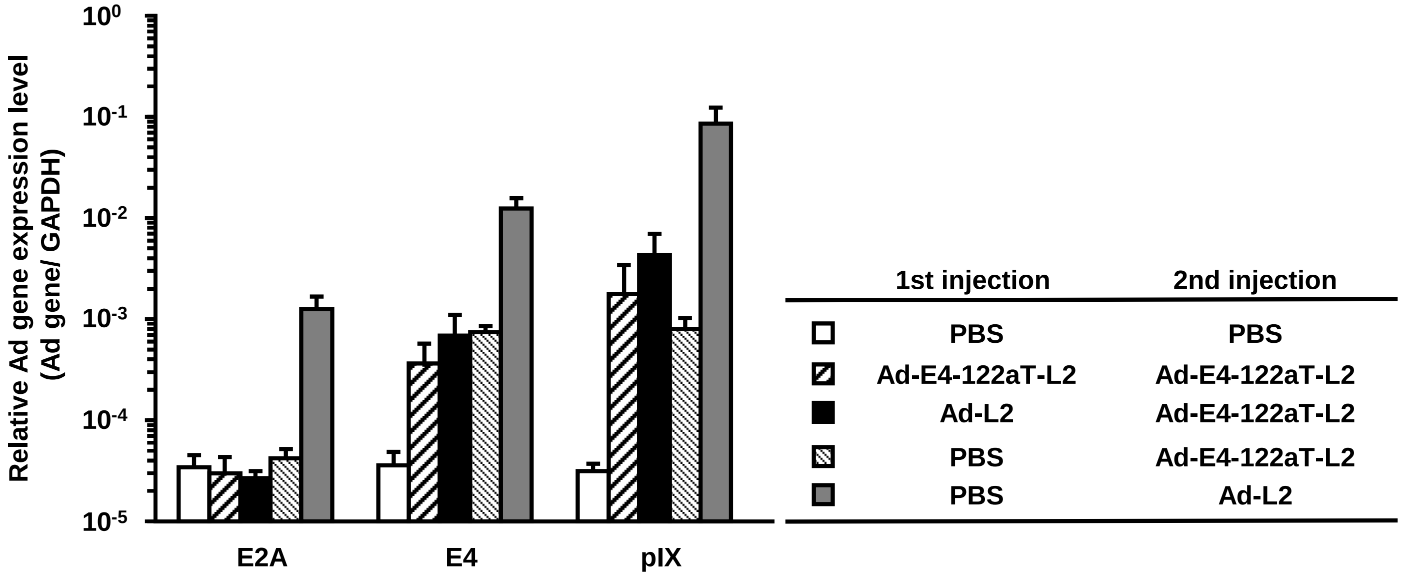

Figure S7. Leaky expression of Ad genes in the liver following sequential administration of Ad vectors. Neonatal mice were administered Ad vectors at a dose of 5.9×10^{11} IFU/kg *via* the retro-orbital sinus on day 0 and *via* the tail vein on day 42. The liver was collected to recover total RNA on day 44 following neonatal injection. Ad gene expression levels were determined by real-time RT-PCR. Results are shown as the averages ± S.D. (n=5-6).

Figure S8

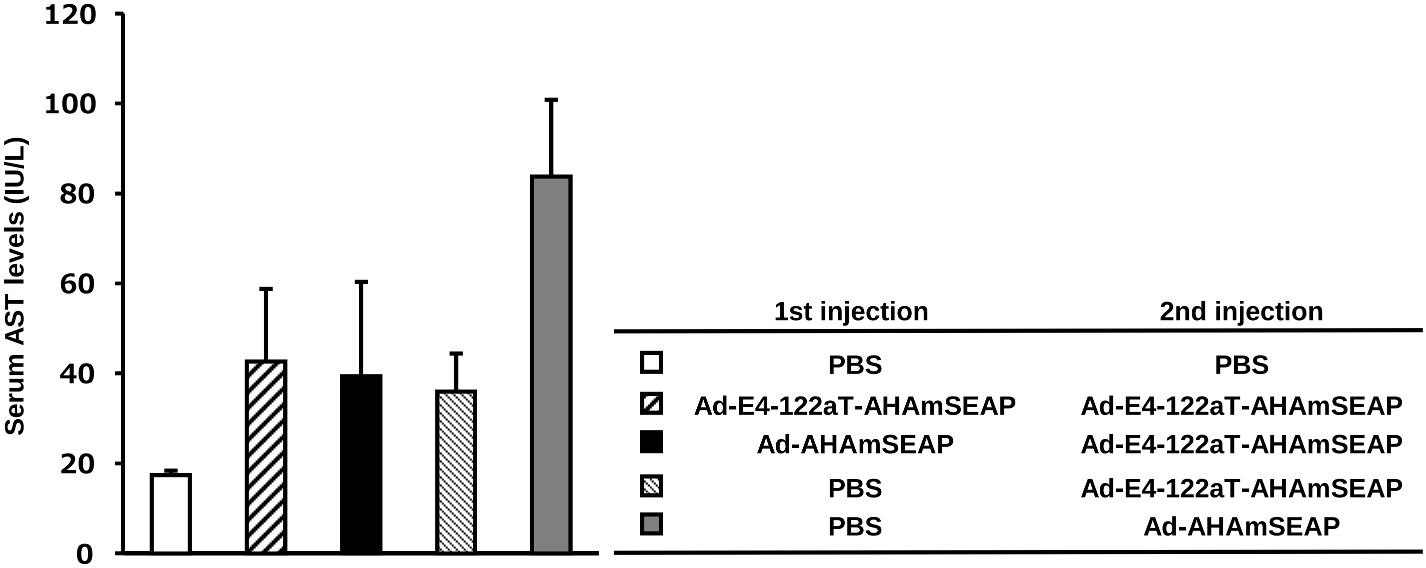

Figure S8. Serum AST levels following sequential administration of Ad vectors. Neonatal mice were administered Ad vectors at a dose of $5.9x10^{11}$ IFU/kg *via* the retro-orbital sinus on day 0 and *via* the tail vein on day 42. The serum was collected on day 49, followed by the measurement of serum AST levels. Results are shown as the averages ± S.D. (n=7). AST, aspartate aminotransferase.

Figure S9

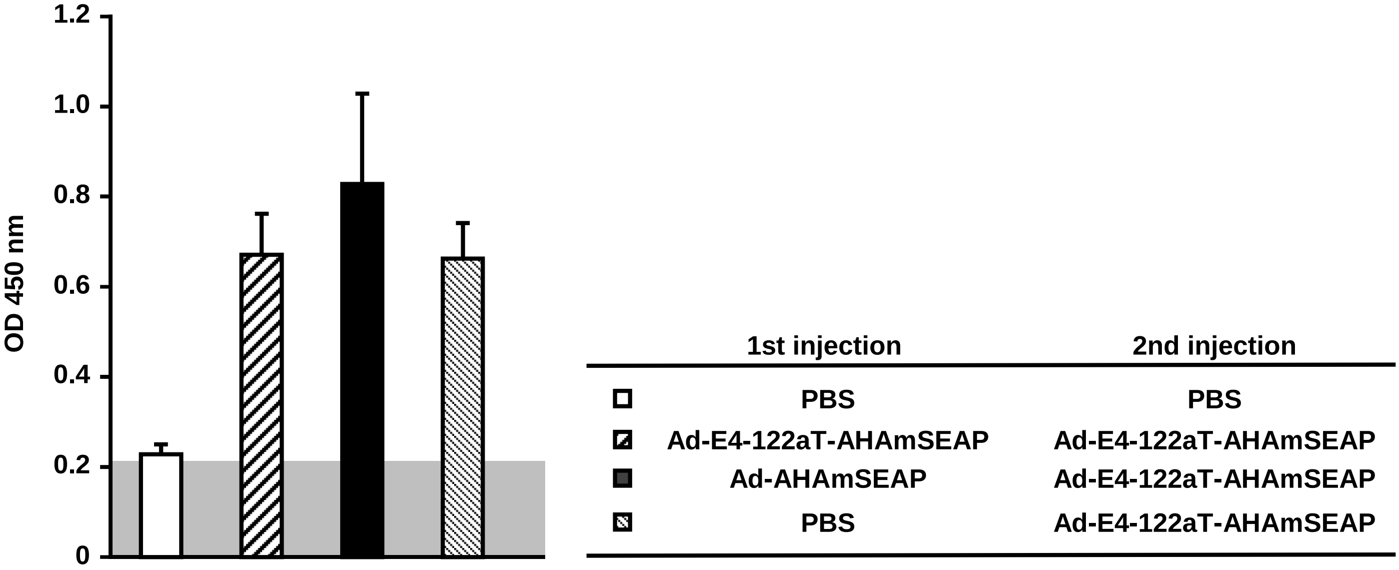

Figure S9. Anti-Ad antibody titers in the mouse serum following sequential administration of Ad vectors. Neonatal mice were administered Ad vectors at a dose of 5.9x10^{11} IFU/kg *via* the retro-orbital sinus on day 0 and *via* the tail vein on day 42. The serum were collected on day 56 following neonatal injection. Anti-Ad antibody titers were determined by ELISA. Serum samples were diluted to 1:2,560. The gray zone represents the background level. Results are shown as the averages ± S.D. (n=7-8).

Figure S10

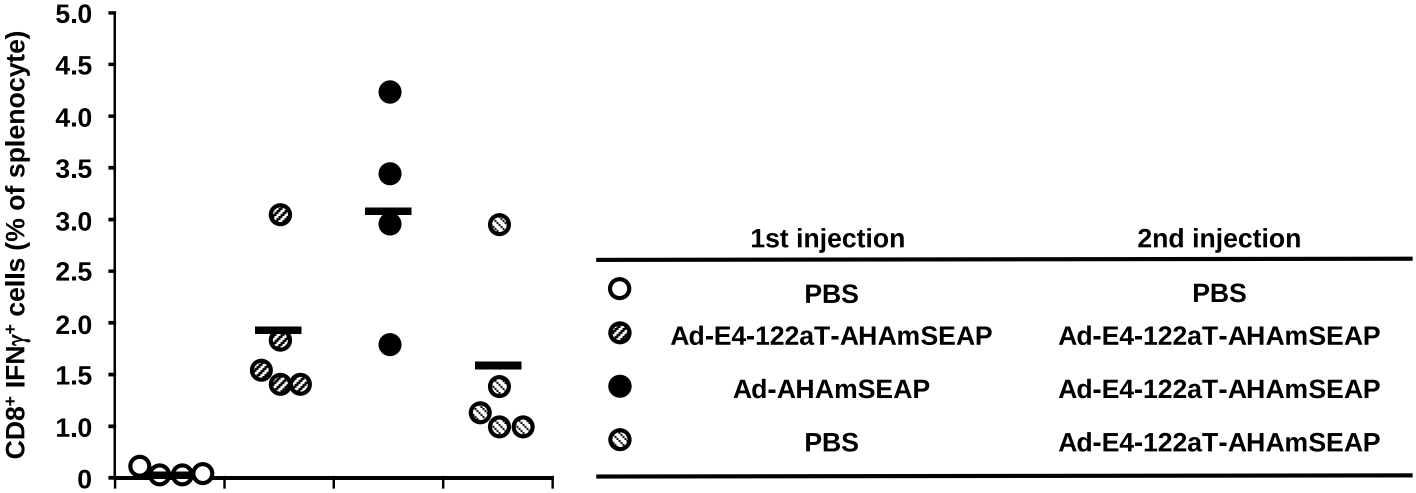

Figure S10. Hexon-specific IFNγ^{+} CD8^{+} T cells in the splenocytes following sequential administration of Ad vectors. Neonatal mice were administered Ad vectors at a dose of 5.9x10^{11} IFU/kg *via* the retro-orbital sinus on day 0 and *via* the tail vein on day 42. The splenocytes were harvested on day 56 following neonatal injection and incubated with hexon peptide for 6 hours. Percentages of IFNγ^{+} CD8^{+} T cells in the splenocytes were detected by FACS. Results are shown as the averages ± S.D. (n=4-5).
