# Supplementary material for: Neonatal Gene Therapy for Hemophilia B by a Novel Adenovirus Vector Showing Reduced Leaky Expression of Viral Genes
Source: Mol Ther Methods Clin Dev. 2017 Jul 8;6:183–93. doi: 10.1016/j.omtm.2017.07.001 (PMC5552065; doi:10.1016/j.omtm.2017.07.001)
Supplement: Document S2. Article plus Supplemental Information [file mmc2.pdf]

Molecular Therapy
Methods & Clinical Development
Original Article

AMERICAN SOCIETY of GENE & CELL THERAPY

Neonatal Gene Therapy for Hemophilia B by a Novel Adenovirus Vector Showing Reduced Leaky Expression of Viral Genes

Shunsuke Iizuka,[1,8] Fuminori Sakurai,[1,2,8] Masashi Tachibana,[1,3] Kazuo Ohashi,[1] and Hiroyuki Mizuguchi[1,4,5,6,7]

[1]Laboratory of Biochemistry and Molecular Biology, Graduate School of Pharmaceutical Sciences, Osaka University, Osaka 565-0871, Japan; [2]Clinical Drug Development Unit, Laboratory of Regulatory Sciences for Oligonucleotide Therapeutics, Graduate School of Pharmaceutical Sciences, Osaka University, Osaka 565-0871, Japan; [3]Laboratory of Biopharmaceutics, Graduate School of Pharmaceutical Sciences, Osaka University, Osaka 565-0871, Japan; [4]iPS Cell-Based Research Project on Hepatic Toxicity and Metabolism, Graduate School of Pharmaceutical Sciences, Osaka University, Osaka 565-0871, Japan; [5]The Center for Advanced Medical Engineering and Informatics, Osaka University, Osaka 565-0871, Japan; [6]Laboratory of Hepatocyte Regulation, National Institute of Biomedical Innovation, Health and Nutrition, Osaka 567-0085, Japan; [7]Global Center for Advanced Medical Engineering and Informatics, Osaka University, Osaka 565-0871, Japan

Gene therapy during neonatal and infant stages is a promising approach for hemophilia B, a congenital disorder caused by deficiency of blood coagulation factor IX (FIX). An adenovirus (Ad) vector has high potential for use in neonatal or infant gene therapy for hemophilia B due to its superior transduction properties; however, leaky expression of Ad genes often reduces the transduction efficiencies by Ad protein-mediated tissue damage. Here, we used a novel Ad vector, Ad-E4-122aT, which exhibits a reduction in the leaky expression of Ad genes in liver, in gene therapy studies for neonatal hemophilia B mice. Ad-E4-122aT exhibited significantly higher transduction efficiencies than a conventional Ad vector in neonatal mice. In neonatal hemophilia B mice, a single neonatal injection of Ad-E4-122aT expressing human FIX (hFIX) (Ad-E4-122aT-AHAFIX) maintained more than 6% of the normal plasma hFIX activity levels for approximately 100 days. Sequential administration of Ad-E4-122aT-AHAFIX resulted in more than 100% of the plasma hFIX activity levels for more than 100 days and rescued the bleeding phenotypes of hemophilia B mice. In addition, immunotolerance to hFIX was induced by Ad-E4-122aT-AHAFIX administration in neonatal hemophilia B mice. These results indicated that Ad-E4-122aT is a promising gene delivery vector for neonatal or infant gene therapy for hemophilia B.

INTRODUCTION

Hemophilia B is an X-linked blood coagulation disorder caused by deficiency or dysfunction of blood coagulation factor IX (FIX) due to genetic mutations in the FIX gene. Patients with hemophilia B exhibit plasma FIX activity levels less than 40% of normal and are divided into three categories according to their plasma FIX activity levels: severe (<1%), moderate (1%–5%), and mild (5%–40%).[1] FIX replacement therapy is often conducted for hemophilia B patients; however, this approach is not always sufficiently therapeutic. In patients with the severe form of the disease, FIX replacement therapy is not curative, and FIX protein should be administered every 2 or 3 days. In addition, immune responses against FIX protein are often induced, especially in severe patients.

However, gene therapy is a promising approach for hemophilia B with the potential to be curative. Favorable therapeutic effects were reported in the clinical trials of gene therapy for hemophilia B.[2] Moreover, gene therapy during the neonatal and infant stages is considered promising for hemophilia B for the following reasons. (1) More than 50% of hemophilia patients are diagnosed in the neonatal period.[3,4] (2) Gene therapy for hemophilia patients during the neonatal and infant stages prevents irreversible symptoms and life-threatening events, such as hemophilic arthropathy and intracranial bleeding. (3) Because neonates have not been exposed to viral vectors, neonates do not possess viral vector-specific immune responses, which often inhibit gene therapy in adults.[5] (4) The lower body weights of neonates allow efficient gene therapy with lower vector doses compared with adults.

Although various types of gene-delivery vehicles, including retrovirus vectors, lentivirus vectors, and adeno-associated virus vectors, have been used in preclinical studies of neonatal gene therapy, adenovirus (Ad) vectors possess various advantages as a gene-delivery vehicle for both adult and neonatal or infant gene therapy.[6–11] We have evaluated the transduction properties of an E1/E3 region-deleted conventional Ad vector based on Ad serotype 5 in neonatal mice.[12] We found

Received 20 February 2017; accepted 5 July 2017;
http://dx.doi.org/10.1016/j.omtm.2017.07.001.

[8]These authors contributed equally to the work.

Correspondence: Fuminori Sakurai, PhD, Laboratory of Biochemistry and Molecular Biology, Graduate School of Pharmaceutical Sciences, Osaka University, 1-6 Yamadaoka, Suita, Osaka, 565-0871, Japan.
E-mail: sakurai@phs.osaka-u.ac.jp
Correspondence: Hiroyuki Mizuguchi, PhD, Laboratory of Biochemistry and Molecular Biology, Graduate School of Pharmaceutical Sciences, Osaka University, 1-6 Yamadaoka, Suita, Osaka, 565-0871, Japan.
E-mail: mizuguch@phs.osaka-u.ac.jp

CrossMark

This is an open access article under the CC BY license (http://creativecommons.org/licenses/by/4.0/).

Table 1. Ad Vectors Used in This Study

Vector Name	Promoter	Transgene	Virus Particles/ Infectious Units
Ad-L2	CMV	Firefly luciferase	9.4
Ad-E4-122aT-L2	CMV	Firefly luciferase	5.69
Ad-AHAmSEAP	AHA	mSEAP	10.51
Ad-E4-122aT-AHA-mSEAP	AHA	mSEAP	8.23
Ad-E4-122aT-AHAFIX	AHA	hFIX	10.10

CMV, cytomegalovirus promoter; AHA, synthetic promoter composed of an apolipoprotein E enhancer, the hepatocyte control region, and a human alpha1-antitrypsin promoter; mSEAP, murine secreted embryonic alkaline phosphatase; hFIX, human factor IX.

that the Ad vector exhibited efficient transduction, especially in the livers of the neonatal mice (second day of life), and because FIX is produced in the hepatocytes, this result suggested that Ad vector-mediated transduction in the neonatal liver would be suitable for hemophilia B gene therapy.[13] However, the transgene expression levels in the neonatal mice were lower than those in the adult mice, and they gradually decreased after administration of a conventional Ad vector. This was attributed to the leaky expression of Ad genes, which has been well established to induce tissue damage, followed by the inhibition of efficient and long-term transgene expression, in adult mice following a conventional Ad vector administration.[14,15] We therefore considered that the leaky expression of Ad genes should be suppressed to achieve efficient and long-term Ad vector-mediated transgene expression in neonates and infants.

To suppress the leaky expression of Ad genes in the liver following Ad vector administration, we developed a novel Ad vector, Ad-E4-122aT.[16] Ad-E4-122aT possesses four copies of sequences perfectly complementary to miR-122a, which consists of approximately 70% of total host microRNAs expressed in the liver in the 3′ UTR of the E4 gene.[17] Ad-E4-122aT exhibited significant suppression of the leaky expression of Ad genes in the liver by miR-122a-mediated suppression of E4 gene expression and significant reduction in the hepatotoxicity and long-term transgene expression in adult mice. In addition, Ad-E4-122aT can be easily grown to high titers via a conventional production method. These results led us to hypothesize that Ad-E4-122aT exhibits safe and efficient transduction profiles in not only adults but also neonates and infants. In this study, therefore, we evaluated the transduction properties of Ad-E4-122aT in neonatal mice and assessed the therapeutic effects of Ad-E4-122aT in neonatal hemophilia B mice. Ad-E4-122aT exhibited higher transduction efficiency in neonatal mice than that of a conventional Ad vector. In addition, Ad-E4-122aT expressing human FIX (hFIX) restored the bleeding phenotype of hemophilia B mice to almost normal levels.

RESULTS

Leaky Expression Levels of Ad Genes in the Neonatal Liver following Systemic Administration of Ad Vectors

To determine whether Ad-E4-122aT suppressed the leaky expression of Ad genes in the neonatal liver, Ad gene expression levels in the liver were determined by real-time RT-PCR analysis following Ad vector administration. The Ad vectors used in this study are described in Table 1 and Figure 1. miR-122a expression levels in the neonatal liver were approximately 50% of those in the adult liver (Figure 2A). Leaky expression of the Ad genes occurred in the neonatal liver following administration of a conventional Ad vector, Ad-L2 (Figure 2B). mRNA levels of the Ad genes in the neonatal livers of Ad-L2-injected mice were about 3.4- to 14.2-fold lower than those in the adult livers of Ad-L2-injected mice (Figure 2B versus Figure S7). The E4 gene expression levels in the livers of Ad-E4-122aT-L2-injected mice were suppressed to 5.4% of those in Ad-L2-injected mice (Figure 2B). The E2A and pIX gene expression levels in the livers of Ad-E4-122aT-L2-injected mice were also suppressed to 1.2% and 4.0% of those in Ad-L2-injected mice. The Ad-E4-122aT-L2-mediated suppression of the Ad gene expression in the neonatal liver was almost comparable to that in the adult liver (Figure 2B; Figure S7). These data indicated that Ad-E4-122aT suppresses the leaky expression of Ad genes in not only the adult liver but also the neonatal liver.

mSEAP Expression in Neonatal Mice following Ad-E4-122aT Administration

To evaluate the transduction efficiencies of Ad-E4-122aT, murine secreted embryonic alkaline phosphatase (mSEAP)-expressing Ad-E4-122aT (Ad-E4-122aT-AHAmSEAP) and a conventional Ad vector (Ad-AHAmSEAP) were systemically administered to neonatal mice. In this experiment, we used mSEAP-expressing Ad vectors, because an mSEAP assay system allows more rigorous evaluation of transduction efficiencies of Ad vectors than an hFIX assay system due to the sensitive and wide measurement range. In Ad-E4-122aT-AHAmSEAP-injected neonatal mice, mSEAP expression levels reached a peak on day 7 and then gradually and slightly decreased from day 7 to day 42 (Figure 3). Significantly higher levels of serum mSEAP were obtained in Ad-E4-122aT-AHAmSEAP-injected mice from day 7 to day 42 compared with Ad-AHAmSEAP-injected mice.

In addition, a second administration of Ad vector was carried out on day 42 following neonatal injection to obtain sustained levels of transgene expression by sequential administration. We previously demonstrated that an Ad vector efficiently transduced the liver following repeated administration in neonatal mice, based on the finding that anti-Ad antibody titers were undetectable in neonatal mice following Ad vector administration.[12] Following repeated administration, mSEAP expression levels were elevated by more than 5-fold compared with those before the second injection (Figure 3). High levels of serum mSEAP persisted for approximately 100 days in the mice receiving Ad-E4-122aT-AHAmSEAP as a second injection, regardless of the types of Ad vectors administered as a first injection to neonatal mice, while the mSEAP expression levels were gradually and significantly decreased in the mice receiving Ad-AHAmSEAP as a second injection. In addition, comparable levels of serum mSEAP levels were found following systemic administration of Ad-E4-122aT-AHAmSEAP in the adult mice with or without neonatal administration of an Ad vector. These data suggested that mice receiving Ad vector administration at the

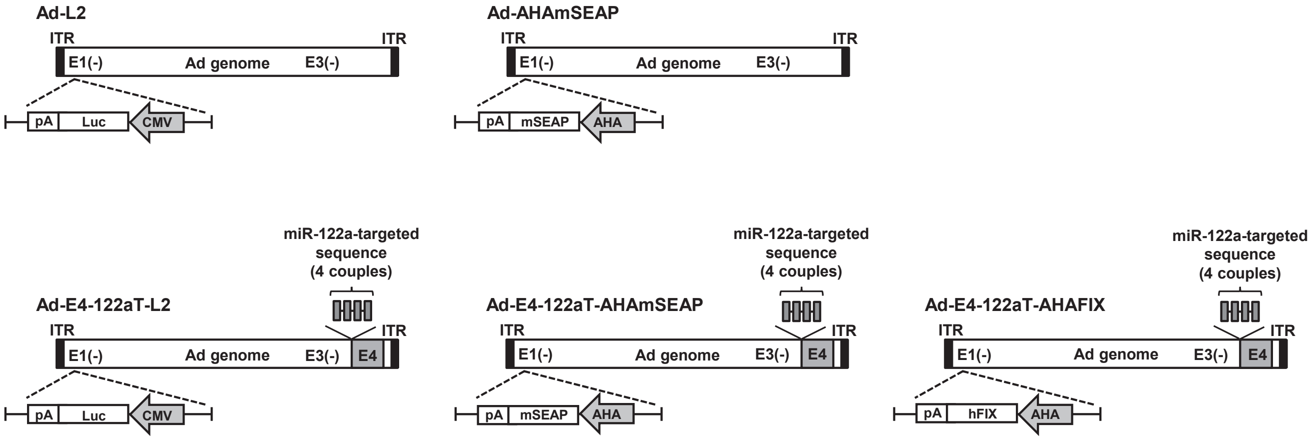

Figure 1. Schematic Diagrams of the Ad Vectors Used in This Study

A luciferase, mSEAP, or hFIX expression cassette was inserted into the E1-deleted region in the Ad vector genome. AHA is a synthetic promoter composed of an apolipoprotein E enhancer, which is the hepatocyte control region, and a human alpha1-antitrypsin promoter. ITR, inverted terminal repeat.

neonatal stage exhibited efficient transgene expression following Ad vector administration at the adult stage and that Ad-E4-122aT exhibited superior transduction profiles following not only neonatal injection but also repeated administration compared with a conventional Ad vector.

Ad Vector Genome Copy Numbers in the Livers of Neonatal Mice following Ad Vector Administration

To assess whether suppression of the leaky expression of Ad genes improved the biodistribution profiles of an Ad vector in neonatal mice, Ad vector genome copy numbers in the livers were determined. On day 2 following Ad vector administration, significant differences in the Ad vector genome copy numbers were not observed in the livers of Ad-E4-122aT-AHAmSEAP- and Ad-AHAmSEAP-injected mice (Figure 4). Ad vector genome copy numbers in the livers were significantly decreased from day 2 to day 6 post-Ad vector administration in Ad-AHAmSEAP-injected mice, while in the livers of Ad-E4-122aT-AHAmSEAP-injected mice, no significant decline in the Ad genome copy numbers was observed from day 2 to day 6 post-administration. In addition, on day 6 following Ad vector administration, Ad-E4-122aT-AHAmSEAP-injected mice exhibited significantly higher Ad vector genome copy numbers in the liver than did Ad-AHAmSEAP-injected mice. These results suggested that Ad vector genomes remained for a longer term in the livers of Ad-E4-122aT-AHAmSEAP-injected neonatal mice than those of Ad-AHAmSEAP-injected mice.

Safety Profiles of Ad-E4-122aT in Neonatal Mice

Innate immune responses and hepatotoxicity were evaluated in neonatal mice to examine the safety profiles of Ad-E4-122aT in neonatal mice following Ad vector administration. mRNA levels of several inflammatory cytokines in the livers and spleens were significantly upregulated 6 hr after neonatal administration of Ad-L2 and Ad-E4-122aT-L2; however, there were no significant differences in the mRNA levels of the inflammatory cytokines between Ad-L2 and Ad-E4-122aT-L2 (Figure S1). These data indicated that similar levels of innate immune responses were induced by Ad-L2 and Ad-E4-122aT-L2.

Serum aspartate aminotransferase (AST) levels, a hepatotoxicity marker, were measured in neonatal mice on day 2 following intravenous administration of Ad-L2 and Ad-E4-122aT-L2 at a dose of 5.9×10^{11} infectious units (IFUs)/kg. No significant differences in the serum AST levels were found among the PBS-, Ad-L2-, and Ad-E4-122aT-L2-injected groups (Figure S2). In addition, we found no significant differences in the body weights of PBS-, Ad-AHAmSEAP-, and Ad-E4-122aT-AHAmSEAP-injected mice following neonatal administration (Figure S3). These data indicated that no apparent hepatotoxicity was induced in neonatal mice following Ad vector administration at this dose. To further examine the hepatotoxicity profiles of Ad-L2 and Ad-E4-122aT-L2 in neonatal mice, Ad vectors were administered to neonatal mice at a dose of 1.8×10^{12} IFU/kg. In the mice receiving Ad-L2 at this dose, significantly higher serum AST levels were observed than in the PBS-injected mice (Figure S2). However, the high dose of Ad-E4-122aT-L2 mediated the serum AST, reducing it to levels comparable to those in PBS-injected mice. In addition, mRNA levels of the albumin gene were significantly lower in the mice receiving the high dose of Ad-L2 than in the mice receiving the high dose of Ad-E4-122aT-L2 on day 2 (Figure S4). Moreover, while all neonatal mice injected with Ad-E4-122aT-L2 survived, several neonatal mice injected with Ad-L2 died within 2 days of administration (Table S1). Leaky expression levels of the Ad genes in the livers of mice receiving the high dose of Ad-E4-122aT-L2 were 5- to 90-fold lower than those in the livers of mice receiving the high dose of Ad-L2 (Figure S5). miR-122a expression levels in the neonatal liver were not altered following administration of Ad-L2 or Ad-E4-122aT-L2 at the dose of 5.9×10^{11} IFU/kg (Figure S6). Collectively, these data indicated that Ad-E4-122aT was a safer vector for neonatal gene therapy than a conventional Ad vector, because the former suppressed Ad protein-mediated hepatotoxicity in neonatal mice.

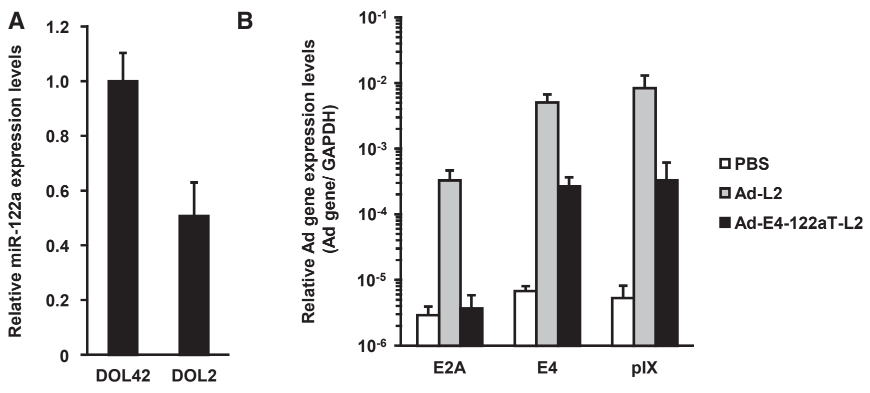

Figure 2. miR-122a-Mediated Suppression of Ad Gene Expression

(A) miR-122a expression levels in adult and neonatal livers. The liver was collected from neonatal and adult mice to recover total RNA. miR-122a expression levels were determined by real-time RT-PCR and normalized by expression levels of U6 snRNA. (B) The leaky expression level of Ad genes in the neonatal mouse liver following systemic administration of Ad vectors. Neonatal mice were administered Ad vectors at a dose of 5.9×10^{11} IFU/kg via retro-orbital sinus. Two days after transduction, the Ad gene expression levels in the liver were determined by real-time RT-PCR. Results are shown as the averages ± SD (n = 5–6). DOL, day of life.

Next, the leaky expression levels of Ad genes and hepatotoxicity levels following second injection of an Ad vector were examined to clarify whether neonatal injection of an Ad vector exacerbated the hepatotoxicities induced by Ad vector administration in the adult stage. The results showed that the mice with and the mice without neonatal administration of an Ad vector exhibited comparable levels of leaky expression of Ad genes in the liver and serum AST following administration of Ad-E4-122aT-AHAmSEAP as a second injection (Figures S7 and S8). These data indicated that neonatal injection of an Ad vector did not augment or suppress the hepatotoxicity of an Ad vector following repeated administration.

We also determined the serum anti-Ad antibody titers and numbers of cytotoxic T lymphocytes (CTLs) against hexon, which is the major Ad capsid protein, in the spleen 14 days following second injection of an Ad vector to examine whether immune responses against Ad proteins were altered by neonatal injection of an Ad vector. The anti-Ad antibody titers in the mice receiving Ad-E4-122aT-AHAmSEAP at the adult stage were comparable between the mice that were neonatally administered an Ad vector and those that were not (Figure S9). Although the average numbers of hexon-specific CTLs were higher in the mice receiving neonatal injection of Ad-AHAmSEAP than in the mice receiving neonatal injection of PBS or Ad-E4-122aT-AHAmSEAP, these differences were not statistically significant (Figure S10). These results appeared to indicate that neonatal injection of an Ad vector did not alter the immune responses against Ad proteins.

hFIX Production in Hemophilia B Mice following Administration of Ad-E4-122aT-AHAFIX

To evaluate the therapeutic effects of hFIX gene transduction in neonatal hemophilia B mice using Ad-E4-122aT, an hFIX-expressing Ad-E4-122aT, Ad-E4-122aT-AHAFIX, was administered to neonatal hemophilia B mice. First, the plasma hFIX antigen and activity levels were measured following the neonatal administration. The plasma hFIX antigen levels on day 14 were 2,351 ± 665 ng/mL (47.0% of the normal level) and gradually decreased to 381 ± 108 ng/mL (7.62% of the normal level) on day 35 (Figure 5A). From day 35 to day 98, Ad-E4-122aT-AHAFIX-injected mice exhibited stable hFIX expression levels ranging from 236 to 496 ng/mL (4.72% to 9.92% of the normal level). The plasma hFIX activity levels were 72.8% and 9.53% of the normal levels on day 14 and day 35, respectively, following Ad-E4-122aT-AHAFIX administration (Figure 5B). From day 35 to day 98, the plasma hFIX activity levels remained stable from 6.3% to 14.3% of the normal levels. Following the second administration of Ad-E4-122aT-AHAFIX, the plasma hFIX antigen and activity levels reached 220%–500% of the normal level (11,000–25,000 ng/mL) and 106%–168% of the normal level, respectively (Figures 5C and 5D). Immunostaining of the liver sections on day 70 revealed that less than 50% of the hepatocytes were hFIX-positive following neonatal single administration of Ad-E4-122aT-AHAFIX and that more than 90% of the hepatocytes expressed hFIX following repeated administration in Ad-E4-122aT-AHAFIX-injected hemophilia B mice (Figure 5E). These data indicated that sufficient plasma hFIX levels were obtained by neonatal administration of Ad-E4-122aT-AHAFIX, based on a report that achieving normal plasma hFIX levels of 1% or more is sufficient for conversion from the severe to the moderate form of hemophilia.[18] In addition, repeated administration of Ad-E4-122aT-AHAFIX significantly elevated the plasma hFIX levels in hemophilia B mice.

Hemophilia Phenotype Correction following Administration of Ad-E4-122aT-AHAFIX

A tail clip assay was carried out on day 70 following neonatal injection of Ad-E4-122aT-AHAFIX to test whether the bleeding phenotype of hemophilia B mice was corrected by Ad-E4-122aT-AHAFIX. The bleeding times of PBS-injected mice were more than 1,500 s following tail clipping (Figure 5F). However, the bleeding times of Ad-E4-122aT-AHAFIX-injected mice decreased to 956 ± 375 s, although one of five mice exhibited bleeding times greater than 1,500 s. In repeatedly Ad-E4-122aT-AHAFIX-injected mice, the bleeding times were 403 ± 122 s, which was shorter than the bleeding times of the wild-type mice (624 ± 167 s) (Figure 5G). These data indicated that Ad-E4-122aT-AHAFIX mediated efficient correction of the bleeding phenotype of hemophilia B mice.

Immune Responses against hFIX in Hemophilia B Mice following Administration of Ad-E4-122aT-AHAFIX

To examine whether immune tolerance to hFIX was induced in hemophilia B mice by neonatal administration of Ad-E4-122aT-AHAFIX,

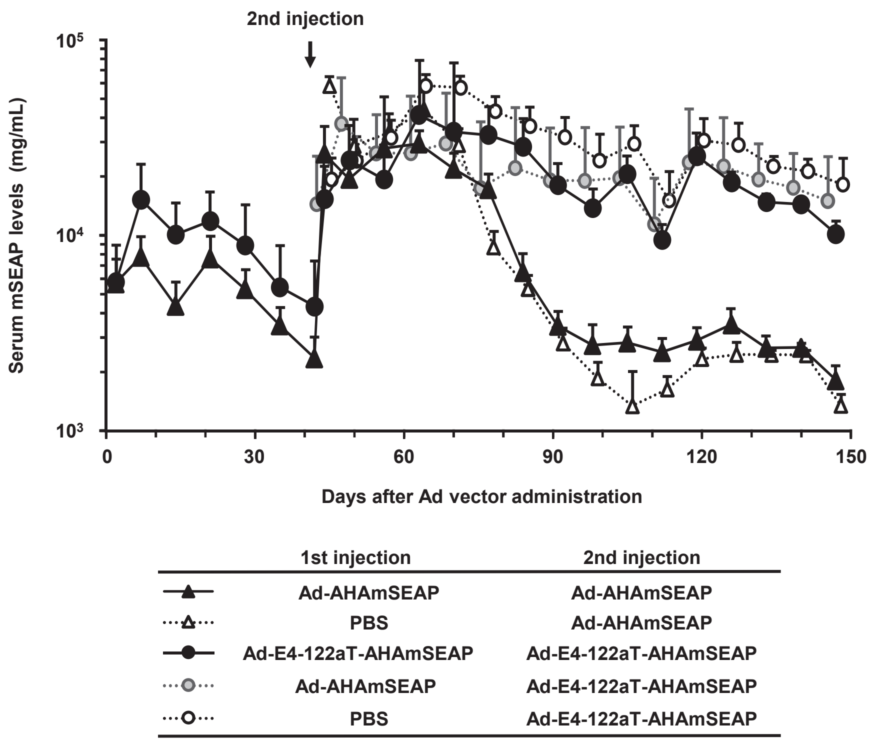

Figure 3. mSEAP Expression Levels following Ad Vector Administration in Neonatal Mice

Neonatal mice were administered mSEAP-expressing Ad vectors at a dose of 5.9×10^{11} IFU/kg via the retro-orbital sinus on day 0 and via the tail vein on day 42. Blood samples were collected via retro-orbital bleeding on the indicated days after injection. mSEAP production in the serum was determined at the indicated time points. Results are shown as the averages ± SD (n = 7–8).

hFIX protein was immunized to hemophilia B mice on day 70 and day 77 following neonatal injection of Ad-E4-122aT-AHAFIX. Before hFIX immunization, anti-hFIX antibody titers were not detectable in either PBS- or Ad-E4-122aT-AHAFIX-injected mice (Figure 6). In PBS-injected mice, high titers of anti-hFIX antibody were detected 2 weeks following immunization with hFIX protein. However, Ad-E4-122aT-AHAFIX-injected mice exhibited almost background levels of anti-hFIX antibody titers following immunization with hFIX. These data indicated that immune tolerance to hFIX was induced in hemophilia B mice by neonatal administration of Ad-E4-122aT-AHAFIX.

DISCUSSION

The aim of this study was to achieve phenotypic correction in hemophilia B mice by sustained expression of hFIX in neonatal mice using a novel Ad vector, Ad-E4-122aT. Ad-E4-122aT possesses four copies of sequences perfectly complementary to miR-122a, which is a liver-specific miRNA, in the 3′ UTR of the E4 gene.[16] We previously demonstrated that Ad-E4-122aT mediated higher levels of transgene expression in adult mice than a conventional Ad vector due to the suppression of Ad protein-mediated hepatotoxicity. Although miR-122a expression levels in the neonatal liver were almost half of those in adults, the Ad gene expression was efficiently knocked down in the neonatal livers of Ad-E4-122aT-injected mice, suggesting that miR-122a expression levels in the neonatal liver were adequate for efficient knockdown of the Ad genes. In this study, Ad-E4-122aT mediated higher levels of transgene expression in neonatal mice compared with a conventional Ad vector. Although no statistically significant differences in the hepatotoxicity levels were detected between Ad-E4-122aT- and conventional Ad vector-injected mice at a dose of 5.9×10^{11} IFU/kg, the conventional Ad vector-injected mice exhibited significantly higher hepatotoxicity levels than the Ad-E4-122aT-injected mice following high dose of Ad vector administration. We consider that because Ad-E4-122aT induced lower levels of hepatotoxicity than the conventional Ad vector, Ad-E4-122aT mediated higher transduction efficiencies than a conventional Ad vector in neonatal mice. In addition, the Ad vector genome copy numbers in the neonatal liver did not significantly decline in Ad-E4-122aT-injected neonatal mice, while apparent reductions in the Ad vector genome copy numbers in the neonatal liver were found in conventional Ad vector-injected neonatal mice.

Conversion from the severe to mild phenotype of hemophilia B dramatically improves quality of life by reducing spontaneous bleeding in patients with severe hemophilia B.[2] Patients with severe hemophilia B require intravenous administration of hFIX every 2–3 days to prevent spontaneous bleeding; however, repeated intravenous injection places a heavy burden on patients, especially neonates and infants.[1]

In this study, a single neonatal injection of Ad-E4-122aT-AHAFIX resulted in plasma hFIX activity levels of greater than 5% in hemophilia B mice, and these levels lasted for at least 98 days, indicating that Ad-E4-122aT-AHAFIX converted the severe phenotype to the mild phenotype. In addition, Ad-E4-122aT-AHAFIX significantly improved the bleeding phenotypes of hemophilia B mice. These results indicated that gene therapy for hemophilia B using Ad-E4-122aT would improve the quality of life for hemophilia B patients.

Ad-E4-122aT-injected hemophilia B mice did not produce detectable levels of anti-hFIX antibody following hFIX immunization, indicating that immune tolerance to hFIX was induced in hemophilia B mice by neonatal gene therapy. Induction of immune tolerance to hFIX in mice following neonatal and fetal administration of hFIX-expressing vector has been well studied.[6,19–23] Previous studies revealed several factors crucial for the induction of immune tolerance to transgene

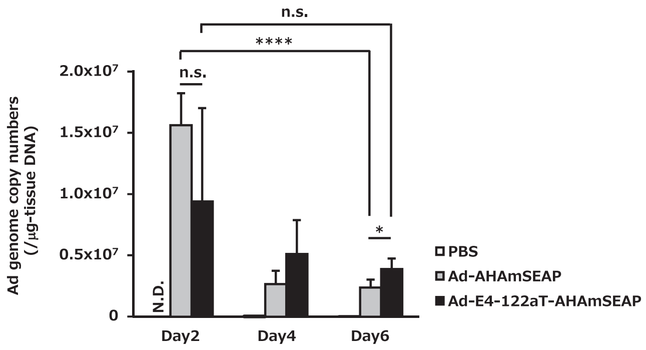

Figure 4. Ad Genome Copy Numbers in the Livers of Neonatal Mice

Neonatal mice were administered Ad vectors at a dose of 5.9×10^{11} IFU/kg via the retro-orbital sinus. Ad genome copy numbers in the liver were examined at the indicated time points. Results are shown as the averages ± SD (n = 4). n.s., not significant. *$p < 0.05$; ****$p < 0.0001$.

products. Shi et al.[19] and Nivsarkar et al.[20] demonstrated the importance of induction of $CD4^+CD25^+$ regulatory T cells. In these studies, induction of anti-hFIX antibodies following hFIX immunization was efficiently suppressed by transfer of $CD4^+CD25^+$ regulatory T cells from mice receiving neonatal or fetal administration of hFIX-expressing vectors. It is also crucial for induction of immune tolerance to transgene products that sufficient and sustained transgene expression be mediated from the neonatal periods. Zhang et al.[21] demonstrated that more than 14 ng/mL (approximately 0.3% of normal levels) of plasma hFIX was required shortly after birth to induce immune tolerance to hFIX. In this study, more than 2,700 ng/mL of hFIX was obtained shortly after neonatal administration of Ad-E4-122aT-AHAFIX, suggesting that Ad-E4-122aT-AHAFIX produced sufficient plasma hFIX antigen levels to induce immune tolerance to hFIX. In addition to high levels of plasma hFIX, liver-specific hFIX expression is considered to contribute to the induction of immune tolerance to hFIX.[24–26] Adeno-associated virus (AAV) vector-mediated liver-specific hFIX expression using an AHA promoter in adult mice efficiently induced immune tolerance to hFIX, while ubiquitous expression of hFIX using a human elongation factor-1 alpha (EF-1α) promoter resulted in efficient induction of immune responses to hFIX.[24] These findings indicated that AHA promoter-mediated liver-specific expression of hFIX contributed to the induction of immune tolerance to hFIX in neonatal hemophilia B mice, although it remains to be determined whether the same is true for neonates.

Although Ad-E4-122aT-AHAmSEAP exhibited higher levels of mSEAP expression than Ad-AHAmSEAP in the neonatal mice, the mSEAP levels gradually declined in both Ad-E4-122aT-AHAmSEAP-treated and Ad-AHAmSEAP-treated mice. This gradual decline was probably due to the growth of the mice, suggesting that repeated administration of an Ad vector is necessary to achieve sustained transgene expression for efficient neonatal gene therapy. We conducted repeated administration of Ad-E4-122aT-AHAmSEAP to restore transgene expression levels in the mice. Following a second injection of Ad-E4-122aT-AHAmSEAP or Ad-E4-122aT-AHAFIX, significant increases in the transgene expression levels and long-term transgene expression were observed. In addition, the transduction profiles of Ad-E4-122aT-AHAmSEAP administered as a second injection in adult mice were not affected by neonatal administration of an Ad vector. These data indicate that efficient and long-term transgene expression can be achieved by repeated administration of Ad-E4-122aT at the neonatal or infant and adult stages.

Immune responses are the major limitation of Ad vector-mediated gene therapy for inherited genetic disorders. Ad hexon-specific CTL was detected in mice following Ad-E4-122aT-L2 administration; however, Ad hexon-specific CTL did not induce apparent hepatotoxicity. We previously demonstrated that the hepatocytes transduced with Ad-E4-122aT were less susceptible to Ad hexon-specific CTL attack than those transduced with a conventional Ad vector due to reduction in the leaky expression of Ad genes in the hepatocytes transduced with Ad-E4-122aT.[16] Inflammatory cytokines were also upregulated following Ad-E4-122aT administration; however, we found that inflammatory cytokine-induced hepatotoxicities, which were observed about 2 days after Ad vector administration, were not detected in neonatal mice. In addition, we previously demonstrated that inflammatory cytokine-induced hepatotoxicities were significantly lower in Ad-E4-122aT-injected adult mice than in conventional Ad vector-injected adult mice. These data suggest that the influences of Ad vector-induced inflammatory cytokines on Ad vector-mediated long-term transgene expression are lower in Ad-E4-122aT-administered mice than in conventional Ad vector-administered mice. In addition, a previous study demonstrated that Ad vector-induced inflammatory responses were suppressed by an anti-inflammatory drug without reduction in the transduction efficiencies of an Ad vector.[27] Co-administration of anti-inflammatory drugs would be a promising approach.

Among the various viral vectors used in the clinical and preclinical studies, AAV vectors are often used for in vivo gene therapy for hemophilia B and other inherited genetic disorders because of their superior transduction profiles.[2,10,11,28,29] Although AAV vectors are favorable for in vivo gene therapy, they possess a few drawbacks. First, the packaging limit of AAV is approximately 5 kb. Thus, larger transgenes—for example, the low-density lipoprotein (LDL) receptor gene (about 5.3 kb), a mutation of which causes familial hypercholesterolemia, or the carbamoyl phosphate synthetase 1 gene (about 5.7 kb), a mutation of which causes carbamoyl phosphate synthetase 1 deficiency—cannot be inserted into the AAV genome. These diseases are important targets for gene therapy. Second, AAV has been reported to cause hepatocellular carcinoma following neonatal administration due to the insertional mutagenesis.[30,31] This novel Ad vector, Ad-E4-122aT, exhibits a safer transduction profile and sustained transgene expression following systemic administration. These findings led us to consider that Ad-E4-122aT would be a promising alternative vector when AAV is not suitable.

Helper-dependent Ad vectors are also a promising tool for neonatal gene therapy due to their superior safety profiles.[32,33] Neonatal

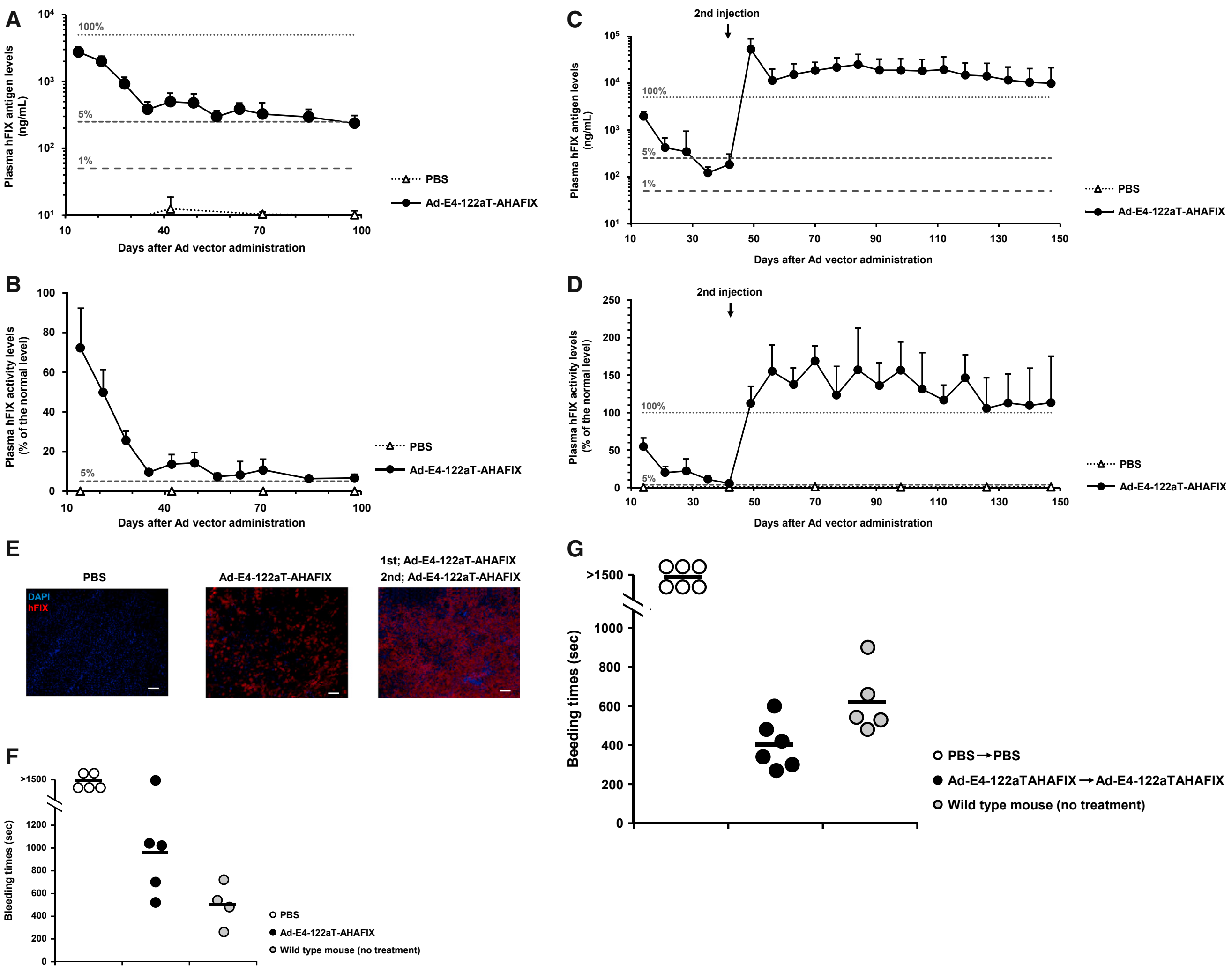

Figure 5. Therapeutic Effects of Ad-E4-122aT-AHAFIX in Neonatal Hemophilia B Mice

Neonatal hemophilia B mice were administered Ad-E4-122aT-AHAFIX at a dose of 5.9×10^{11} IFU/kg via the retro-orbital sinus on day 0. In mice in the repeated administration group, a second injection was conducted on day 42 via the tail vein. (A and B) Plasma hFIX antigen (A) and activity (B) levels following single administration. (C and D) Plasma hFIX antigen (C) and activity (D) levels following repeated administration. (E) Immunohistochemical staining of the frozen liver sections. The liver was recovered on day 70 following neonatal injection. Scale bar, 100 μm. (F and G) Bleeding times of hemophilia B mice following tail clipping. Mouse tails were clipped on day 70. Bleeding times were measured following single administration (F) and repeated administration (G). Results are shown as the averages ± SD (n = 4–7).

administration of a helper-dependent Ad vector expressing factor VIII in hemophilia A mice resulted in sustained expression of factor VIII and efficient therapeutic effects; however, the low titer production and cumbersome production procedure of helper-dependent Ad vectors are problematic.[34] Still, high titers of Ad-E4-122aT can be prepared via a conventional vector preparation procedure. Altogether, these results show that Ad-E4-122aT is a promising alternative for a helper-dependent Ad vector for neonatal gene therapy, although a larger transgene could be inserted into a helper-dependent Ad vector compared with Ad-E4-122aT.

In this study, we conducted neonatal gene therapy for hemophilia B mice using a novel Ad vector that exhibited a reduction in leaky expression of Ad genes, Ad-E4-122aT. Ad-E4-122aT exhibited higher transduction efficiencies in neonatal mice compared with a conventional Ad vector. In addition, repeated administration of Ad-E4-122aT resulted in high and stable transgene expression. Ad-E4-122aT-AHAFIX achieved sufficient levels of plasma hFIX in neonatal mice and restored the bleeding phenotype of hemophilia B. These results indicate that Ad-E4-122aT is a promising vector for hemophilia B gene therapy during the neonatal and infant stages. We should pay close attention to the differences in transduction profiles of Ad vectors between human and rodents. In a clinical trial of hepatic artery infusion of an Ad vector, transgene expression levels in the human liver were lower than those expected from the data in a mouse model; however, a clinical study

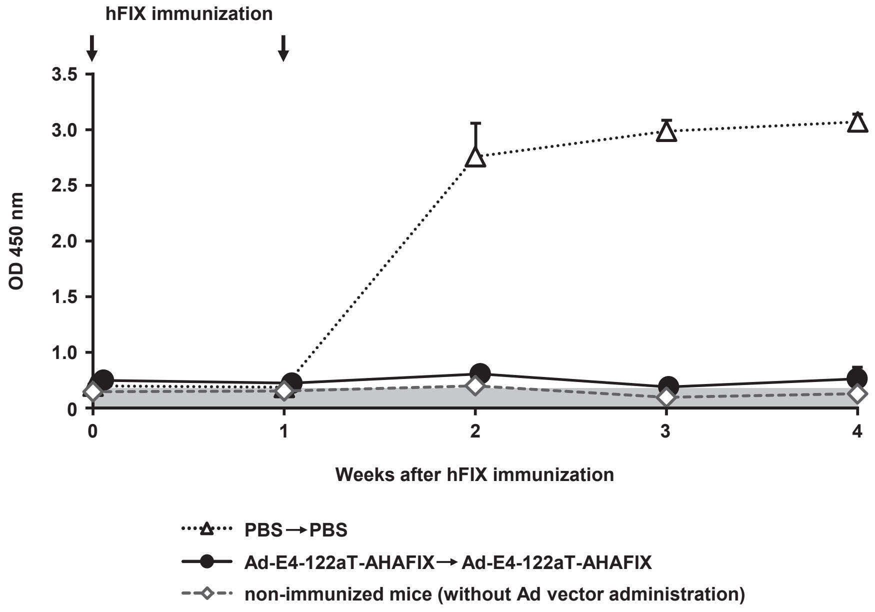

Figure 6. Anti-hFIX Antibody Production in hFIX Immunized-Hemophilia B Mice

Neonatal hemophilia B mice were administered Ad-E4-122aT-AHAFIX at a dose of 5.9×10^{11} IFU/kg via the retro-orbital sinus on day 0 and via the tail vein on day 42. Hemophilia B mice were immunized with hFIX in CFA on days 70 and 77. Non-immunized mice were subcutaneously injected with PBS in CFA on days 70 and 77. Plasma anti-hFIX antibody titers were measured by ELISA at the indicated time points. Plasma samples were diluted to 1:2,560. The gray zone represents the background level. Results are shown as the averages ± SD (n = 5–6).

reported that the highest Ad genome copy numbers among the organs examined were detected in the liver, indicating that the liver is also the main target organ of Ad vectors in humans.[35,36] The liver is the most important target organ for the treatment of congenital disorders. This study demonstrates the therapeutic potential of Ad-E4-122aT-AHAFIX for neonatal gene therapy using neonatal hemophilia B mice. Ad-E4-122aT is a promising vector for numerous congenital disorders in which the therapeutic gene is too large to fit into an AAV vector.

MATERIALS AND METHODS

Cell Culture

HEK293 cells (a human embryonic kidney cell line) were cultured in DMEM (Wako Pure Chemical) supplemented with 10% fetal calf serum (FCS), 2 mM glutamine, and antibiotics.

Plasmids and Replication-Incompetent Ad Vectors

All Ad vectors used in this study were based on Ad serotype 5. A first-generation Ad vector containing a cytomegalovirus (CMV) promoter-driven firefly luciferase-expressing cassette (Ad-L2) and a synthetic liver-specific AHA promoter-driven mSEAP-expressing cassette (Ad-AHAmSEAP) were previously constructed.[16,37,38] The AHA promoter was composed of an apolipoprotein E enhancer, which is the hepatocyte control region, and a human alpha1-antitrypsin promoter.

Ad-E4-122aT containing a CMV promoter-driven firefly luciferase-expressing cassette (Ad-E4-122aT-L2) and an AHA promoter-driven mSEAP-expressing cassette (Ad-E4-122aT-AHAmSEAP) was previously constructed.[16] Ad-E4-122aT containing an AHA promoter-driven hFIX-expressing cassette (Ad-E4-122aT-AHAFIX) was prepared by an improved in vitro ligation method as described later.[39,40] Briefly, an AHA promoter-driven hFIX-expressing plasmid (pAHA-hFIX) was constructed using pHMRSV, pBS-ApoEHCR-hAATp-hFIX-Int-bpA, and pCMV-FIX-ZSGreen1.[38,40] pAHA-hFIX was digested by I-*Ceu*I/PI-*Sce*I and subsequently ligated with I-*Ceu*I/PI-*Sce*I-digested pAdHM4-E4-122aT, resulting in pAd-E4-122aT-AHAFIX. pAd-E4-122aT-AHAFIX was digested with *Pac*I to release the recombinant viral genome and was transfected into HEK293 cells plated on 60-mm dishes. Ad vectors were propagated in HEK293 cells, purified by two rounds of cesium chloride-gradient ultracentrifugation, dialyzed, and stored at −80°C. The virus particles (VPs) were quantified using a spectrophotometric method.[41] Biological titers were measured using an Adeno-X-rapid titer kit (Clontech). The ratios of the particle-to-infectious titer were between 5.6 and 14.5 for each Ad vector used in this study. The Ad vectors used in this study are listed in Table 1 and Figure 1.

Mice and Animal Experimental Procedures

C57BL/6 mice aged 6 weeks were obtained from Nippon SLC. FIX knockout mice were purchased from Jackson Laboratory. Second day-of-life (DOL) mice were used as the neonatal animals. Female mice aged 6 weeks were used as the adult animals. Ad vectors were injected at doses of 5.9×10^{11}–2.9×10^{12} IFU/kg via the retro-orbital sinus of neonatal mice and via the tail vein of adult mice.[42] The body weights of neonatal and adult mice were approximately 1.7 and 17 g, respectively. An Ad vector injection was performed in a total volume of 20 μL in neonatal mice and 200 μL in adult mice. Blood samples were collected by retro-orbital bleeding in adult mice or venous bleeding in neonatal mice. All animal experimental procedures used in this study were performed in accordance with the institutional guidelines for animal experiments at Osaka University.

Real-Time RT-PCR Analysis of miR-122a and Ad Gene Expression Levels

Total RNA was extracted from the liver using ISOGEN II (Wako Pure Chemical) for miR-122a expression analysis. Reverse transcription was performed using a TaqMan MicroRNA Reverse Transcription Kit specific for has-miR-122a (Applied Biosystems). miR-122a

expression levels were determined by using a TaqMan Real-time RT-PCR system and normalized by the U6 small nuclear RNA (snRNA) expression levels.

Total RNA was extracted from the liver using ISOGEN (Wako Pure Chemicals) 2 days after administration of Ad-L2 and Ad-E4-122aT-L2 for Ad gene expression analysis in the liver. Ad gene expression levels were determined by TaqMan real-time PCR systems as previously described.[43]

Real-Time PCR Analysis of Ad Vector Genome Copy Numbers in the Liver

Total DNA, including the Ad vector genome, was extracted from the livers of mice receiving Ad-AHAmSEAP or Ad-E4-122aT-AHAmSEAP using a DNeasy Blood and Tissue Kit (QIAGEN). Ad vector genome copy numbers in the liver were evaluated by real-time PCR analysis as previously described.[44,45]

Detection of mSEAP Expression Levels in the Serum

Ad-AHAmSEAP and Ad-E4-122aT-AHAmSEAP were administered to neonatal mice via the retro-orbital sinus. On day 42 after the first injection, Ad vectors were sequentially administered via the tail vein. Blood samples were collected at the indicated time points and centrifuged (4°C, 7,000 × *g*, 15 min) to obtain the serum. The mSEAP expression levels in the serum were determined using a Great EscAPe SEAP Chemiluminescence Kit v.2.0 (Clontech), according to the manufacturer's instructions.

Determination of Plasma hFIX Levels in Hemophilia B Mice

Ad-E4-122aT-AHAFIX was administered on day 0 (single administration) or on both day 0 and day 42 (repeated administration) to neonatal hemophilia B mice. Blood samples were collected on the indicated days and immediately anticoagulated with 0.1 vol of 3.8% sodium citrate (Fuso Pharmaceutical Industries), followed by centrifugation (4°C, 7,000 × *g*, 5 min) to obtain the plasma. The plasma hFIX antigen levels were measured using a Human Factor IX ELISA Kit (Assaypro). hFIX activity levels were measured using a KC4 Delta Coagulometer (Trinity Biotech). In brief, 50 μL of the plasma samples were 10-fold diluted; mixed with 50 μL of an hFIX-deficient plasma, ThromboCheck FIX (Sysmex), and 50 μL of an activated partial thromboplastin time (APTT) reagent, ThromboCheck APTT-SLA (Sysmex); and then incubated for 3 min at 37°C, followed by addition of 50 μL of 0.02 M $CaCl_2$ (Sysmex). The standard curve was created using a 10-fold diluted normal human plasma, Coagtrol N (Sysmex).

Evaluation of Phenotypic Correction in Hemophilia B Mice

Phenotypic correction was evaluated by measuring the bleeding times following tail clipping. On day 70 following neonatal injection, the mouse tail was clipped from 5 mm of the tip. Bleeding times were monitored using filter paper. The cutoff time was 1,500 s (25 min).

Immunostaining of the Frozen Liver Sections

The liver lobes were harvested on day 70 following neonatal injection of Ad-E4-122aT-AHAFIX from hemophilia B mice receiving single or repeated administration, mounted in optimal cutting temperature (OCT) compound (Sakura) and frozen in Cryomolds (Sakura) with liquid nitrogen. Frozen tissues were sectioned at 10 μm thickness and mounted onto silane-coated slides (Dako). Fluorescent staining was performed as previously described using goat anti-hFIX polyclonal antibody (GAFIX-AP, 1:100) (Affinity Biologicals) and donkey anti-goat immunoglobulin G (IgG) Alexa Flour 594 (A-11058, 1:1,000) (Life Technologies).[46]

Analysis of anti-hFIX Antibody Titers

Ad-E4-122aT-AHAFIX was administered on day 0 and day 42 to neonatal hemophilia B mice. On day 70 following neonatal injection, mice were immunized by subcutaneous injection of 5 μg of hFIX protein (Sigma-Aldrich) in complete Freund's adjuvant (CFA) (Difco Laboratories). On day 77, the second immunization was performed. The plasma samples were collected, followed by measurement of anti-hFIX antibody titers by ELISA as previously described.[16] A 96-well immune plate (Thermo Fisher Scientific) was pre-coated with 0.1 μg of hFIX/well.

Statistical Analysis

All results are shown as the mean ± SD of the mean. Statistical significance was analyzed by one-way ANOVA followed by Bonferroni multiple comparison test using GraphPad Prism 5.04 software (GraphPad).

SUPPLEMENTAL INFORMATION

Supplemental Information includes Supplemental Materials and Methods, ten figures, and one table and can be found with this article online at http://dx.doi.org/10.1016/j.omtm.2017.07.001.

AUTHOR CONTRIBUTIONS

S.I. designed and performed the experiments, analyzed data, and wrote the manuscript; F.S. designed and supervised the projects, analyzed data, and wrote the manuscript; M.T. analyzed data and supervised the projects; and K.O. and H.M. supervised the projects, interpreted data, and wrote the manuscript.

CONFLICTS OF INTEREST

The authors declare no conflict of interest regarding the publication of this paper.

ACKNOWLEDGMENTS

We thank Sayuri Okamoto and Eri Hosoyamada (Osaka University, Osaka, Japan) for their help. We also thank Kohei Tatsumi (Kinki University, Osaka Japan) for providing technical suggestions. The AHA promoter was a gift of Mark A. Kay (Stanford University, Stanford, CA). The hFIX gene was provided by Tomohiko Yamazaki (International Center for Materials Nanoarchitectonics, National Institute for Materials Science, Tsukuba, Japan). This study was supported by a Grant-in-Aid for Scientific Research (A) and (C) and a Grant-in-Aid for Young Scientists (A) from the Ministry of Education, Culture, Sports, Sciences, and Technology of Japan and Bayer Hemophilia Award Program. S.I. is supported by a

Grant-in-Aid for the Japan Society for the Promotion of Science (JSPS) fellows.

REFERENCES

1. Srivastava, A., Brewer, A.K., Mauser-Bunschoten, E.P., Key, N.S., Kitchen, S., Llinas, A., Ludlam, C.A., Mahlangu, J.N., Mulder, K., Poon, M.C., and Street, A.; Treatment Guidelines Working Group on Behalf of The World Federation Of Hemophilia (2013). Guidelines for the management of hemophilia. Haemophilia *19*, e1–e47.
2. Nathwani, A.C., Reiss, U.M., Tuddenham, E.G.D., Rosales, C., Chowdary, P., McIntosh, J., Della Peruta, M., Lheriteau, E., Patel, N., Raj, D., et al. (2014). Long-term safety and efficacy of factor IX gene therapy in hemophilia B. N. Engl. J. Med. *371*, 1994–2004.
3. Conway, J.H., and Hilgartner, M.W. (1994). Initial presentations of pediatric hemophiliacs. Arch. Pediatr. Adolesc. Med. *148*, 589–594.
4. Chambost, H., Gaboulaud, V., Coatmélec, B., Rafowicz, A., Schneider, P., and Calvez, T.; Suivi thérapeutique National des Hémophiles (SNH) Group (2002). What factors influence the age at diagnosis of hemophilia? Results of the French hemophilia cohort. J. Pediatr. *141*, 548–552.
5. Manno, C.S., Pierce, G.F., Arruda, V.R., Glader, B., Ragni, M., Rasko, J.J.E., Ozelo, M.C., Hoots, K., Blatt, P., Konkle, B., et al. (2006). Successful transduction of liver in hemophilia by AAV-Factor IX and limitations imposed by the host immune response. Nat. Med. *12*, 342–347.
6. Xu, L., Gao, C., Sands, M.S., Cai, S.R., Nichols, T.C., Bellinger, D.A., Raymer, R.A., McCorquodale, S., and Ponder, K.P. (2003). Neonatal or hepatocyte growth factor-potentiated adult gene therapy with a retroviral vector results in therapeutic levels of canine factor IX for hemophilia B. Blood *101*, 3924–3932.
7. Traas, A.M., Wang, P., Ma, X., Tittiger, M., Schaller, L., O'donnell, P., Sleeper, M.M., Vite, C., Herati, R., Aguirre, G.D., et al. (2007). Correction of clinical manifestations of canine mucopolysaccharidosis I with neonatal retroviral vector gene therapy. Mol. Ther. *15*, 1423–1431.
8. Nguyen, T.H., Bellodi-Privato, M., Aubert, D., Pichard, V., Myara, A., Trono, D., and Ferry, N. (2005). Therapeutic lentivirus-mediated neonatal in vivo gene therapy in hyperbilirubinemic Gunn rats. Mol. Ther. *12*, 852–859.
9. Kobayashi, H., Carbonaro, D., Pepper, K., Petersen, D., Ge, S., Jackson, H., Shimada, H., Moats, R., and Kohn, D.B. (2005). Neonatal gene therapy of MPS I mice by intravenous injection of a lentiviral vector. Mol. Ther. *11*, 776–789.
10. Hu, C., and Lipshutz, G.S. (2012). AAV-based neonatal gene therapy for hemophilia A: long-term correction and avoidance of immune responses in mice. Gene Ther. *19*, 1166–1176.
11. Yang, Y., Wang, L., Bell, P., McMenamin, D., He, Z., White, J., Yu, H., Xu, C., Morizono, H., Musunuru, K., et al. (2016). A dual AAV system enables the Cas9-mediated correction of a metabolic liver disease in newborn mice. Nat. Biotechnol. *34*, 334–338.
12. Iizuka, S., Sakurai, F., Shimizu, K., Ohashi, K., Nakamura, S., Tachibana, M., and Mizuguchi, H. (2015). Evaluation of transduction properties of an adenovirus vector in neonatal mice. BioMed Res. Int. *2015*, 685374.
13. Boost, K.A., Auth, M.K.H., Woitaschek, D., Kim, H.-S., Hilgard, P., Nadalin, S., and Blaheta, R.A. (2007). Long-term production of major coagulation factors and inhibitors by primary human hepatocytes in vitro: perspectives for clinical application. Liver Int. *27*, 832–844.
14. Yang, Y., Ertl, H.C.J., and Wilson, J.M. (1994). MHC class I-restricted cytotoxic T lymphocytes to viral antigens destroy hepatocytes in mice infected with E1-deleted recombinant adenoviruses. Immunity *1*, 433–442.
15. Yang, Y., Nunes, F.A., Berencsi, K., Furth, E.E., Gönczöl, E., and Wilson, J.M. (1994). Cellular immunity to viral antigens limits E1-deleted adenoviruses for gene therapy. Proc. Natl. Acad. Sci. USA *91*, 4407–4411.
16. Shimizu, K., Sakurai, F., Tomita, K., Nagamoto, Y., Nakamura, S., Katayama, K., Tachibana, M., Kawabata, K., and Mizuguchi, H. (2014). Suppression of leaky expression of adenovirus genes by insertion of microRNA-targeted sequences in the replication-incompetent adenovirus vector genome. Mol. Ther. Methods Clin. Dev. *1*, 14035.
17. Lagos-Quintana, M., Rauhut, R., Yalcin, A., Meyer, J., Lendeckel, W., and Tuschl, T. (2002). Identification of tissue-specific microRNAs from mouse. Curr. Biol. *12*, 735–739.
18. Kay, M.A., and High, K. (1999). Gene therapy for the hemophilias. Proc. Natl. Acad. Sci. USA *96*, 9973–9975.
19. Shi, Y., Falahati, R., Zhang, J., Flebbe-Rehwaldt, L., and Gaensler, K.M. (2013). Role of antigen-specific regulatory CD4+CD25+ T cells in tolerance induction after neonatal IP administration of AAV-hF.IX. Gene Ther. *20*, 987–996.
20. Nivsarkar, M.S., Buckley, S.M.K., Parker, A.L., Perocheau, D., McKay, T.R., Rahim, A.A., Howe, S.J., and Waddington, S.N. (2015). Evidence for contribution of CD4+ CD25+ regulatory T cells in maintaining immune tolerance to human factor IX following perinatal adenovirus vector delivery. J. Immunol. Res. *2015*, 397879.
21. Zhang, J., Xu, L., Haskins, M.E., and Parker Ponder, K. (2004). Neonatal gene transfer with a retroviral vector results in tolerance to human factor IX in mice and dogs. Blood *103*, 143–151.
22. Waddington, S.N., Nivsarkar, M.S., Mistry, A.R., Buckley, S.M.K., Kemball-Cook, G., Mosley, K.L., Mitrophanous, K., Radcliffe, P., Holder, M.V., Brittan, M., et al. (2004). Permanent phenotypic correction of hemophilia B in immunocompetent mice by prenatal gene therapy. Blood *104*, 2714–2721.
23. Waddington, S.N., Buckley, S.M.K., Nivsarkar, M., Jezzard, S., Schneider, H., Dahse, T., Kemball-Cook, G., Miah, M., Tucker, N., Dallman, M.J., et al. (2003). In utero gene transfer of human factor IX to fetal mice can induce postnatal tolerance of the exogenous clotting factor. Blood *101*, 1359–1366.
24. Mingozzi, F., Liu, Y.-L., Dobrzynski, E., Kaufhold, A., Liu, J.H., Wang, Y., Arruda, V.R., High, K.A., and Herzog, R.W. (2003). Induction of immune tolerance to coagulation factor IX antigen by in vivo hepatic gene transfer. J. Clin. Invest. *111*, 1347–1356.
25. Cao, O., Dobrzynski, E., Wang, L., Nayak, S., Mingle, B., Terhorst, C., and Herzog, R.W. (2007). Induction and role of regulatory CD4+CD25+ T cells in tolerance to the transgene product following hepatic in vivo gene transfer. Blood *110*, 1132–1140.
26. Cooper, M., Nayak, S., Hoffman, B.E., Terhorst, C., Cao, O., and Herzog, R.W. (2009). Improved induction of immune tolerance to factor IX by hepatic AAV-8 gene transfer. Hum. Gene Ther. *20*, 767–776.
27. Seregin, S.S., Appledorn, D.M., McBride, A.J., Schuldt, N.J., Aldhamen, Y.A., Voss, T., Wei, J., Bujold, M., Nance, W., Godbehere, S., and Amalfitano, A. (2009). Transient pretreatment with glucocorticoid ablates innate toxicity of systemically delivered adenoviral vectors without reducing efficacy. Mol. Ther. *17*, 685–696.
28. Maguire, A.M., Simonelli, F., Pierce, E.A., Pugh, E.N., Jr., Mingozzi, F., Bennicelli, J., Banfi, S., Marshall, K.A., Testa, F., Surace, E.M., et al. (2008). Safety and efficacy of gene transfer for Leber's congenital amaurosis. N. Engl. J. Med. *358*, 2240–2248.
29. Gaudet, D., Méthot, J., Déry, S., Brisson, D., Essiembre, C., Tremblay, G., Tremblay, K., de Wal, J., Twisk, J., van den Bulk, N., et al. (2013). Efficacy and long-term safety of alipogene tiparvovec (AAV1-LPLS447X) gene therapy for lipoprotein lipase deficiency: an open-label trial. Gene Ther. *20*, 361–369.
30. Chandler, R.J., LaFave, M.C., Varshney, G.K., Trivedi, N.S., Carrillo-Carrasco, N., Senac, J.S., Wu, W., Hoffmann, V., Elkahloun, A.G., Burgess, S.M., and Venditti, C.P. (2015). Vector design influences hepatic genotoxicity after adeno-associated virus gene therapy. J. Clin. Invest. *125*, 870–880.
31. Donsante, A., Miller, D.G., Li, Y., Vogler, C., Brunt, E.M., Russell, D.W., and Sands, M.S. (2007). AAV vector integration sites in mouse hepatocellular carcinoma. Science *317*, 477.
32. Alba, R., Bosch, A., and Chillon, M. (2005). Gutless adenovirus: last-generation adenovirus for gene therapy. Gene Ther. *12* (*Suppl 1*), S18–S27.
33. Mian, A., Guenther, M., Finegold, M., Ng, P., Rodgers, J., and Lee, B. (2005). Toxicity and adaptive immune response to intracellular transgenes delivered by helper-dependent vs. first generation adenoviral vectors. Mol. Genet. Metab. *84*, 278–288.
34. Hu, C., Cela, R.G., Suzuki, M., Lee, B., and Lipshutz, G.S. (2011). Neonatal helper-dependent adenoviral vector gene therapy mediates correction of hemophilia A and tolerance to human factor VIII. Proc. Natl. Acad. Sci. USA *108*, 2082–2087.
35. Raper, S.E., Yudkoff, M., Chirmule, N., Gao, G.-P., Nunes, F., Haskal, Z.J., Furth, E.E., Propert, K.J., Robinson, M.B., Magosin, S., et al. (2002). A pilot study of in vivo liver-directed gene transfer with an adenoviral vector in partial ornithine transcarbamylase deficiency. Hum. Gene Ther. *13*, 163–175.
36. Raper, S.E., Chirmule, N., Lee, F.S., Wivel, N.A., Bagg, A., Gao, G.P., Wilson, J.M., and Batshaw, M.L. (2003). Fatal systemic inflammatory response syndrome in a ornithine transcarbamylase deficient patient following adenoviral gene transfer. Mol. Genet. Metab. *80*, 148–158.

37. Mizuguchi, H., Koizumi, N., Hosono, T., Utoguchi, N., Watanabe, Y., Kay, M.A., and Hayakawa, T. (2001). A simplified system for constructing recombinant adenoviral vectors containing heterologous peptides in the HI loop of their fiber knob. Gene Ther. *8*, 730–735.

38. Miao, C.H., Ohashi, K., Patijn, G.A., Meuse, L., Ye, X., Thompson, A.R., and Kay, M.A. (2000). Inclusion of the hepatic locus control region, an intron, and untranslated region increases and stabilizes hepatic factor IX gene expression in vivo but not in vitro. Mol. Ther. *1*, 522–532.

39. Mizuguchi, H., and Kay, M.A. (1998). Efficient construction of a recombinant adenovirus vector by an improved in vitro ligation method. Hum. Gene Ther. *9*, 2577–2583.

40. Mizuguchi, H., and Kay, M.A. (1999). A simple method for constructing E1- and E1/E4-deleted recombinant adenoviral vectors. Hum. Gene Ther. *10*, 2013–2017.

41. Maizel, J.V., Jr., White, D.O., and Scharff, M.D. (1968). The polypeptides of adenovirus. I. Evidence for multiple protein components in the virion and a comparison of types 2, 7A, and 12. Virology *36*, 115–125.

42. Yardeni, T., Eckhaus, M., Morris, H.D., Huizing, M., and Hoogstraten-Miller, S. (2011). Retro-orbital injections in mice. Lab Anim. (NY) *40*, 155–160.

43. Shimizu, K., Sakurai, F., Machitani, M., Katayama, K., and Mizuguchi, H. (2011). Quantitative analysis of the leaky expression of adenovirus genes in cells transduced with a replication-incompetent adenovirus vector. Mol. Pharm. *8*, 1430–1435.

44. Xu, Z.L., Mizuguchi, H., Ishii-Watabe, A., Uchida, E., Mayumi, T., and Hayakawa, T. (2001). Optimization of transcriptional regulatory elements for constructing plasmid vectors. Gene *272*, 149–156.

45. Koizumi, N., Kawabata, K., Sakurai, F., Watanabe, Y., Hayakawa, T., and Mizuguchi, H. (2006). Modified adenoviral vectors ablated for coxsackievirus-adenovirus receptor, alphav integrin, and heparan sulfate binding reduce in vivo tissue transduction and toxicity. Hum. Gene Ther. *17*, 264–279.

46. Rogers, G.L., and Hoffman, B.E. (2012). Optimal immunofluorescent staining for human factor IX and infiltrating T cells following gene therapy for hemophilia B. J. Genet. Syndr. Gene Ther. *S1*, 1–16.

OMTM, Volume *6*

Supplemental Information

Neonatal Gene Therapy for Hemophilia B by a Novel Adenovirus Vector Showing Reduced Leaky Expression of Viral Genes

Shunsuke Iizuka, Fuminori Sakurai, Masashi Tachibana, Kazuo Ohashi, and Hiroyuki Mizuguchi

Supplementary materials and methods

Analysis of serum hepatotoxicity marker

Blood samples were collected and centrifuged (4°C, 7,000 g, 15 min) to obtain the serum. The serum aspartateaminotransferase (AST) levels were determined using a transaminase-CII kit (Wako Pure Chemical).

Real-time RT PCR analysis

Total RNA was extracted from the liver and spleen using ISOGEN (Wako Pure Chemical, Osaka, Japan). mRNA levels of cytokines, including interleukin-(IL-) 6, IL-12, interferon (IFN) -γ, and glyceroaldehyde-3-phosphatedehydrogenase (GAPDH), and albumin were determined by real-time RT-PCR using THUNDERBIRD SYBR qPCR Mix (Toyobo, Osaka, Japan), as previously described [1].

Detection of anti-Ad antibody

Ad-AHAmSEAP and Ad-E4-122aT-AHAmSEAP were administered to neonatal mice *via* the retro-orbital sinus. On day 42 after the 1st injection, Ad vectors were sequentially administered *via* the tail vein. Serum samples were collected as described above on day 56 following neonatal administration. Anti-Ad antibody titers were determined by ELISA as previously described [2]. 96-well immune-plate (Thermo Fisher Scientific, Waltham, MA) was pre-coated with $5x10^8$ vp of Ad-null/well. Serum samples were diluted to 1:2,560.

Anlysis of Hexon specific CTL responses

Ad-AHAmSEAP and Ad-E4-122aT-AHAmSEAP were administered to neonatal mice *via* the retro-orbital sinus. On day 42 after the 1st injection, Ad vectors were sequentially administered *via* the tail vein. Splenocytes were harvested on day 56 following 1st injection. Ad hexon specific CTLs in the splenocytes were examined using a Cytofix/CytoPerm Plus kit (BD Biosciences, San Diego, CA). Briefly, $2x10^6$ splenocytes were cultured with Ad hexon peptide pool (Milteny Biotec, Bergisch Gladbach, Germany), co-stimulation antibody (anti-mouse CD28 and CD49d; eBioscience, San Diego, CA) and GolgiStop (BD Biosciences) in RPMI-1640 (Sigma-Aldrich, St. Louis, MO). Six hours after incubation, splenocytes were stained for viability using Live/ Dead Fixable Dead Cell Stain Kits (Invitrogen, Carlsbad, CA) for 30 min at room temperature. Following incubation and washing with PBS, splenocytes were stained with phycoerythrin (PE)-conjugated anti-mouse CD3ε antibody (eBioscience) and allophycocyanin (APC)-Cy7-conjugated anti-mouse CD8 antibody (Biolegend, San Diego, CA) for 30 min at 4°C. After incubation and washing with PBS, splenocytes were incubated in Cytofix/Cytoperm solution for 30 min at 4°C for permeabilization and stained with PE-Cy7-conjugated anti-mouse interferon (IFN)-γ antibody (eBioscience). Data were analyzed using FlowJo software (TreeStar, Ashland, OR).

Supplementary references

[1] S. Iizuka, F. Sakurai, K. Shimizu, K. Ohashi, S.-I. Nakamura, M. Tachibana, H. Mizuguchi, Evaluation of transduction properties of an adenovirus vector in neonatal mice, Biomed Res. Int. 2015 (2015). doi:10.1155/2015/685374.

[2] K. Shimizu, F. Sakurai, K. Tomita, Y. Nagamoto, S. Nakamura, K. Katayama, M. Tachibana, K. Kawabata, H. Mizuguchi, Suppression of leaky expression of adenovirus genes by insertion of microRNA-targeted sequences in the replication-incompetent adenovirus vector genome, Mol. Ther. — Methods Clin. Dev. 1 (2014) 14035. doi:10.1038/mtm.2014.35.

Figure S1

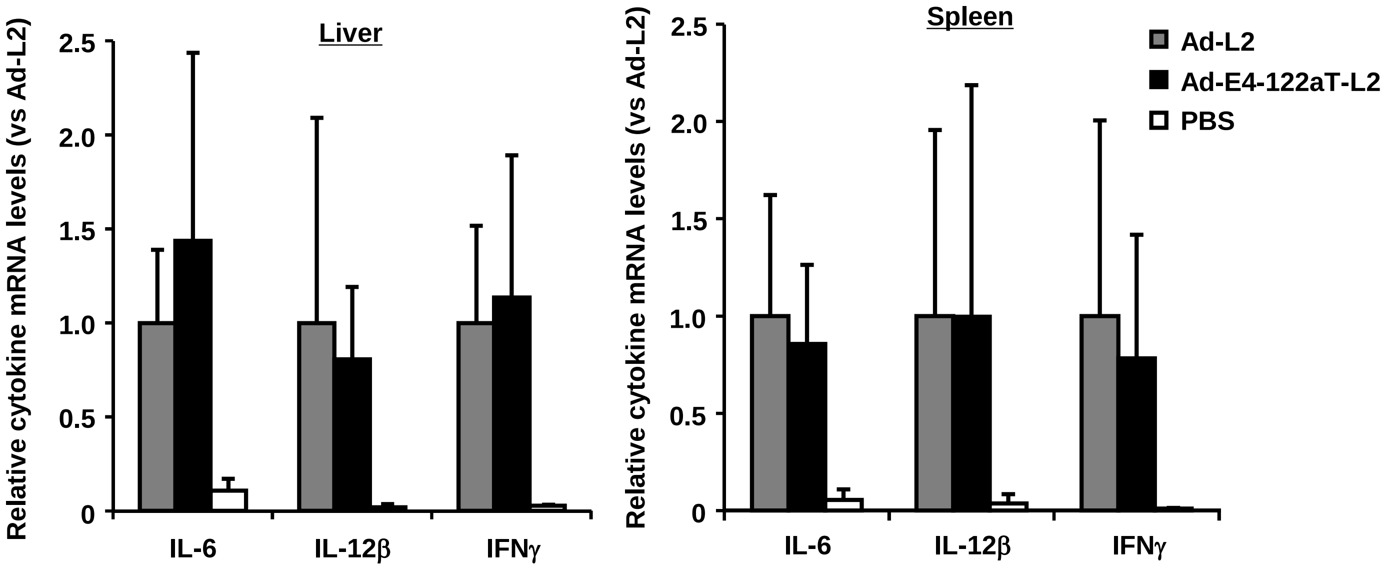

Figure S1. Innate immune responses in the neonatal liver and spleen following systemic administration of Ad vectors. Neonatal mice were administered Ad vectors at a dose of 5.9×10^{11} IFU/kg *via* the retro-orbital sinus. The liver and spleen were collected 6 h after Ad vector administration to recover total RNA. All values were normalized to 1 by the data from the Ad-L2-injected group. Results are shown as the averages ± S.D. (n=5-6).

Figure S2

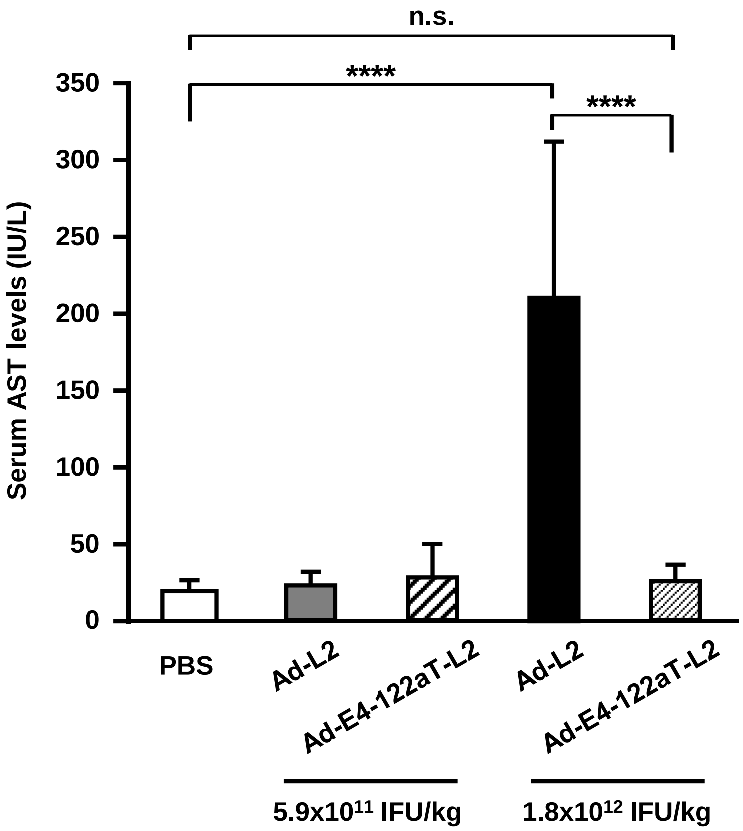

Figure S2. Serum AST levels following Ad vector administration in neonatal mice. Neonatal mice were administered Ad vectors at a dose of 5.9×10^{11} IFU/kg or 1.8×10^{12} IFU/kg *via* the retro-orbital sinus. On day 2 following Ad vector administration, serum AST levels were measured. Results are shown as the averages ± S.D. (n=7-8). AST, aspartate aminotransferase; n.s., not significant. ****$p < 0.0001$.

Figure S3

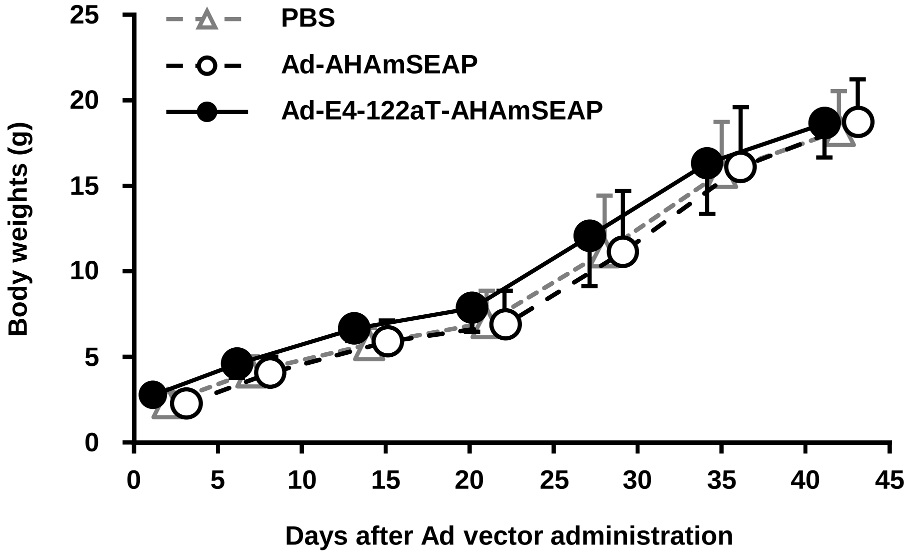

Figure S3. Mouse body weights following Ad vector administration in neonatal mice. Neonatal mice were administered Ad vectors at a dose of 5.9×10^{11} IFU/kg *via* the retro-orbital sinus. Body weights were measured at the indicated time points. Results are shown as the averages ± S.D. (n=7-8).

Figure S4

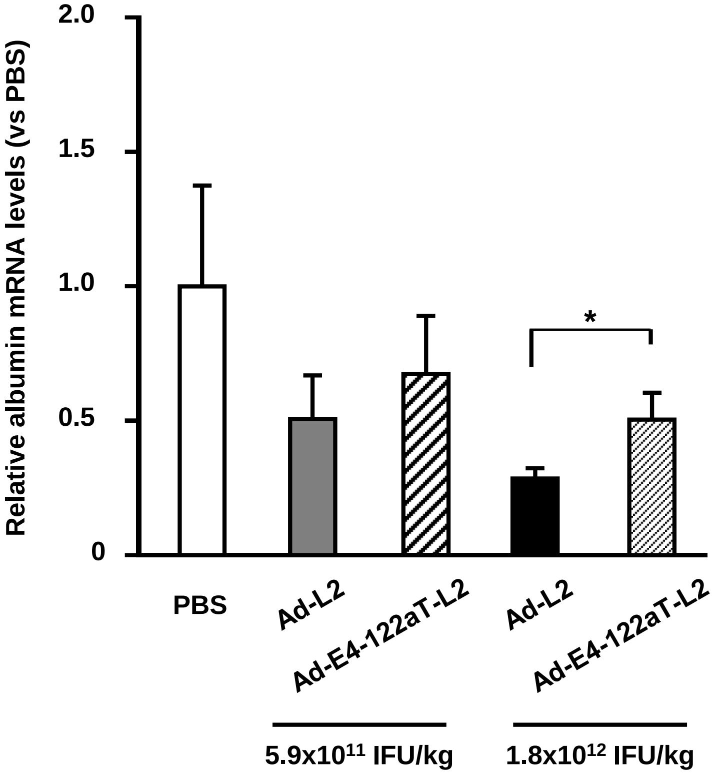

Figure S4. Endogenous albumin expression levels in the neonatal liver following systemic administration of Ad vectors. Neonatal mice were administered Ad vectors at a dose of 5.9×10^{11} IFU/kg or 1.8×10^{12} IFU/kg *via* the retro-orbital sinus. The liver was harvested to recover total RNA on day 2 following Ad vector administration. Endogenous albumin expression levels were measured by real-time RT-PCR. All values were normalized to 1 by the data from the PBS-injected group. Results are shown as the averages ± S.D. (n=6). n.s., not significant. *$p < 0.05$. ** $p < 0.01$.

Table S1

Table S1. Viability of neonatal mice following systemic administration of high doses of Ad vectors.

Injection titer	*Ad vector*	*Number of injected mice*	*Number of dead mice*	*Viability*
$1.8x10^{12}$ IFU/kg	Ad-L2	9	2	77.8%
	Ad-E4-122aT-L2	9	0	100%
$2.9x10^{12}$ IFU/kg	Ad-L2	6	3	50%
	Ad-E4-122aT-L2	6	0	100%

Neonatal mice were administered Ad vectors at a dose of $1.8x10^{12}$ IFU/kg or $2.9x10^{12}$ IFU/kg *via* the retro-orbital sinus. Two days after transduction, viabilities of mice were examined.

Figure S5

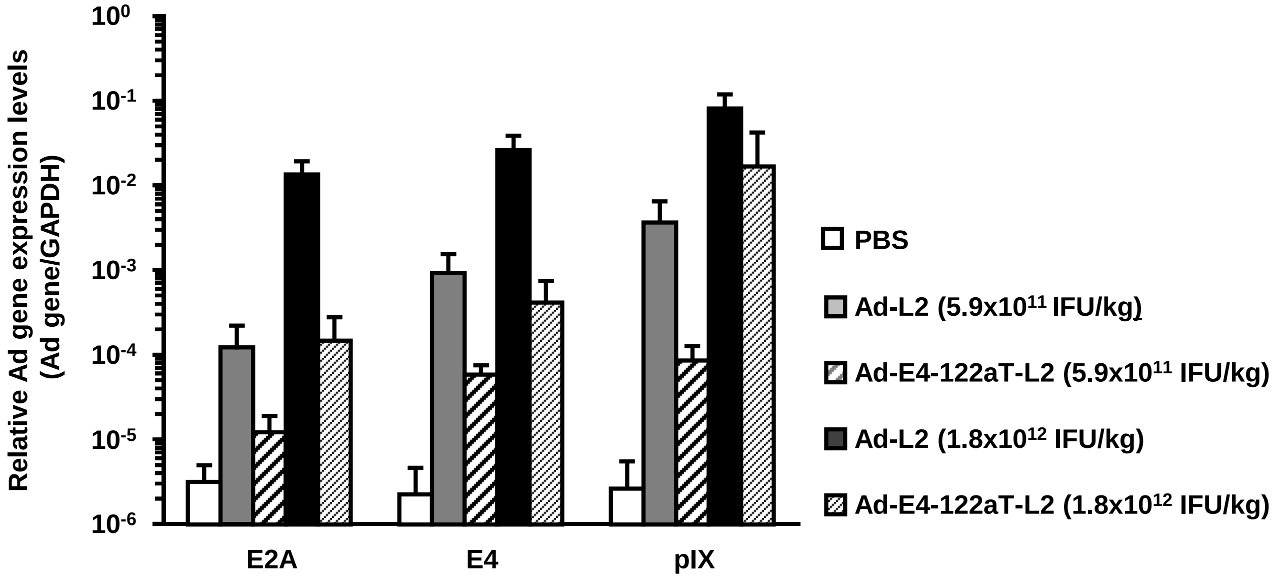

Figure S5. Leaky expression of Ad genes in the neonatal mouse liver following systemic administration of Ad vectors. Neonatal mice were administered Ad vectors at a dose of 5.9×10^{11} IFU/kg or 1.8×10^{12} IFU/kg *via* the retro-orbital sinus. Two days after transduction, the Ad gene expression levels in the liver were determined by real-time RT-PCR. Results are shown as the averages ± S.D. (n=5-6).

Figure S6

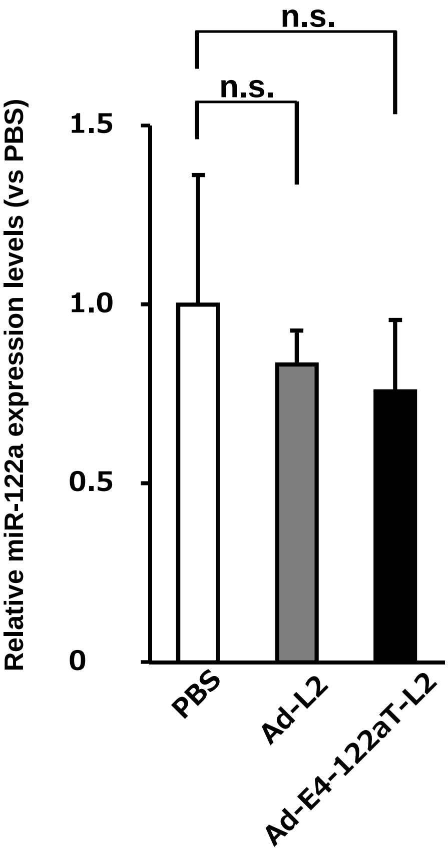

Figure S6. miR-122a expression levels in the neonatal liver following systemic administration of Ad vectors. Neonatal mice were administered Ad vectors at a dose of 5.9×10^{11} IFU/kg *via* the retro-orbital sinus. The liver was harvested to recover total RNA on day 2 following Ad vector administration. miR-122a expression levels were measured by real-time RT-PCR. All values were normalized to 1 by the data from the PBS-injected group. Results are shown as the averages ± S.D. (n=6). n.s., not significant.

Figure S7

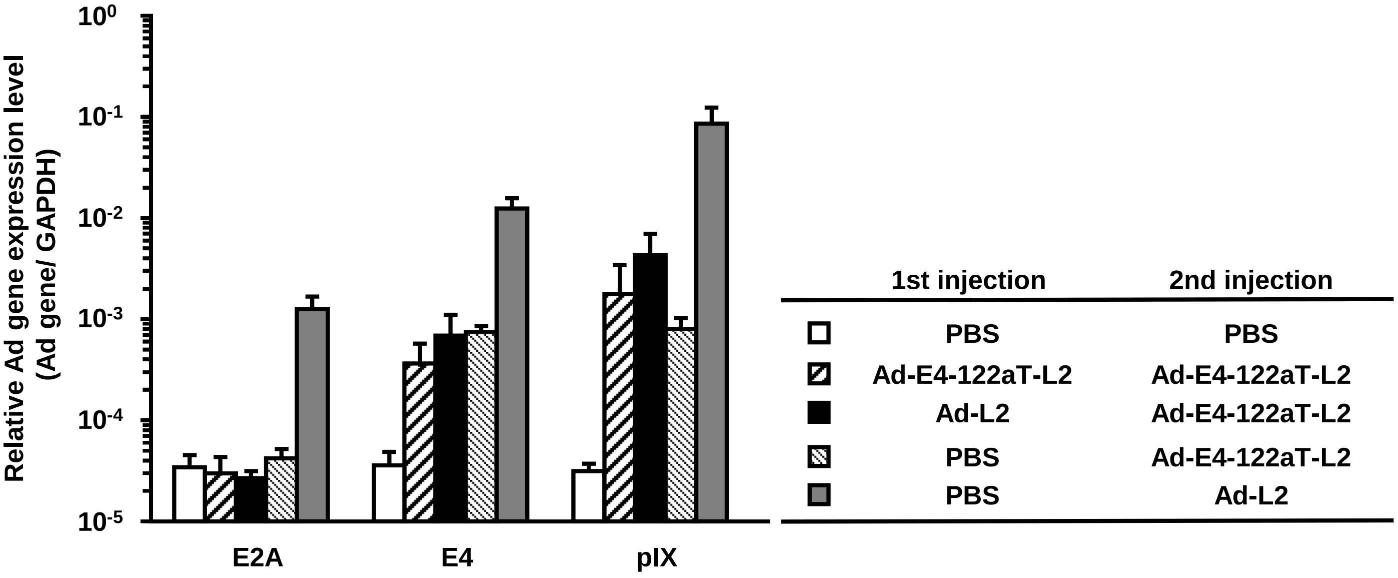

Figure S7. Leaky expression of Ad genes in the liver following sequential administration of Ad vectors. Neonatal mice were administered Ad vectors at a dose of 5.9×10^{11} IFU/kg *via* the retro-orbital sinus on day 0 and *via* the tail vein on day 42. The liver was collected to recover total RNA on day 44 following neonatal injection. Ad gene expression levels were determined by real-time RT-PCR. Results are shown as the averages ± S.D. (n=5-6).

Figure S8

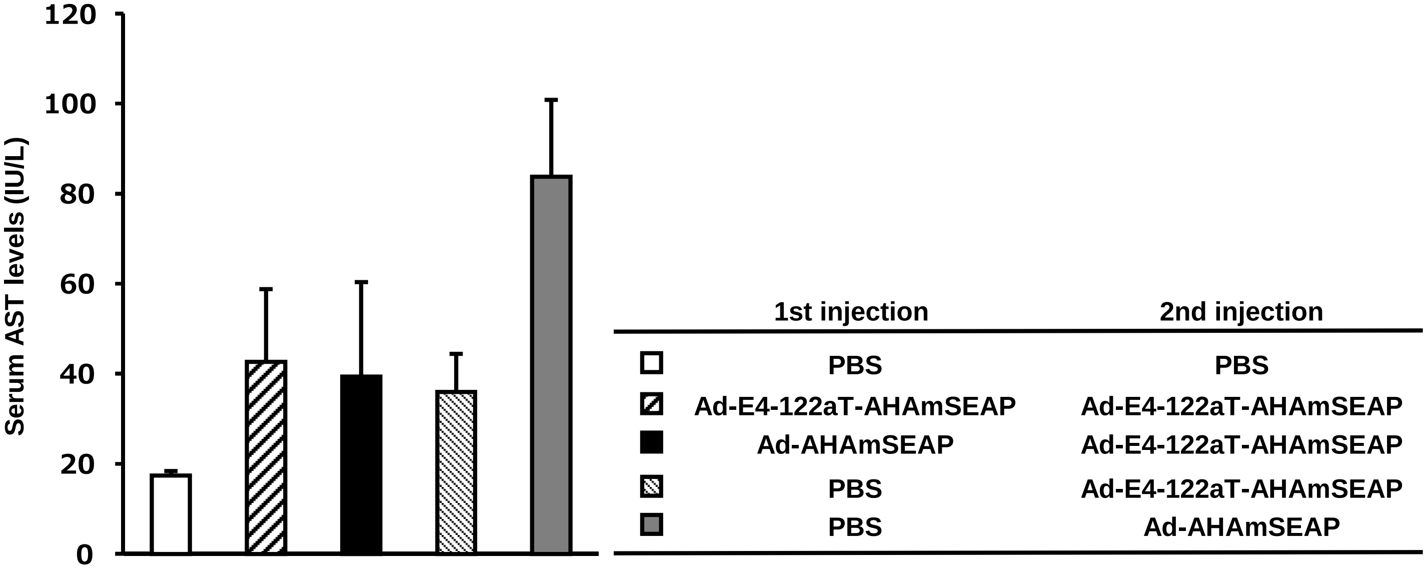

Figure S8. Serum AST levels following sequential administration of Ad vectors. Neonatal mice were administered Ad vectors at a dose of 5.9×10^{11} IFU/kg *via* the retro-orbital sinus on day 0 and *via* the tail vein on day 42. The serum was collected on day 49, followed by the measurement of serum AST levels. Results are shown as the averages ± S.D. (n=7). AST, aspartate aminotransferase.

Figure S9

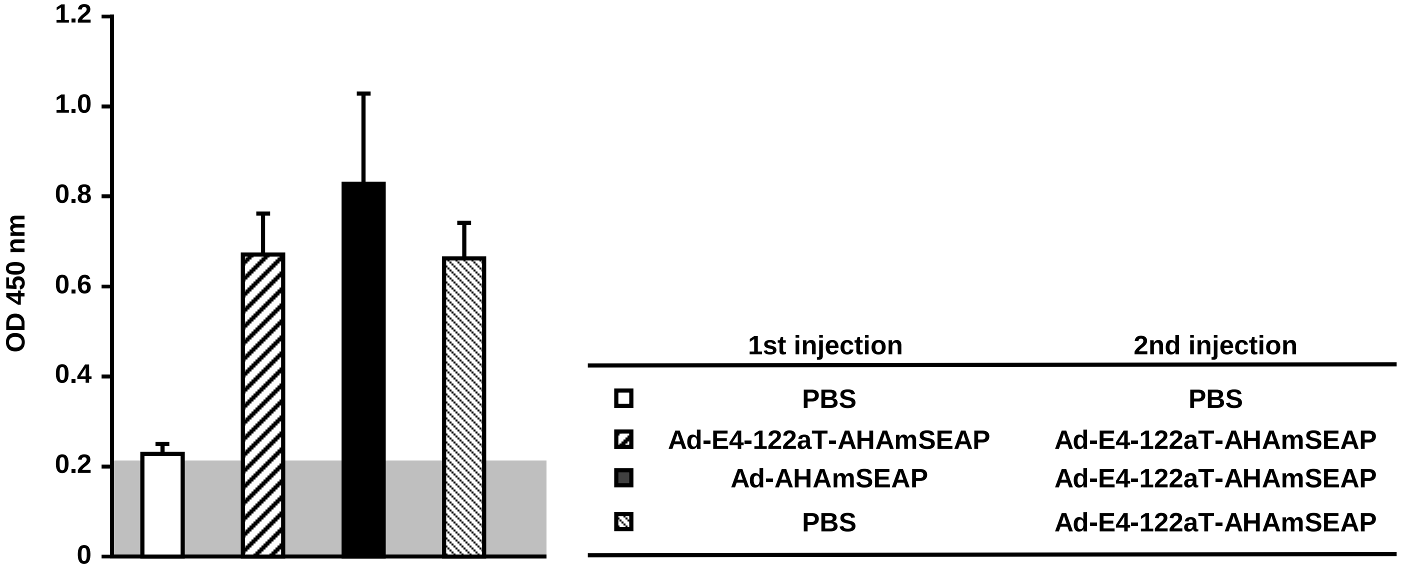

Figure S9. Anti-Ad antibody titers in the mouse serum following sequential administration of Ad vectors. Neonatal mice were administered Ad vectors at a dose of 5.9×10^{11} IFU/kg *via* the retro-orbital sinus on day 0 and *via* the tail vein on day 42. The serum were collected on day 56 following neonatal injection. Anti-Ad antibody titers were determined by ELISA. Serum samples were diluted to 1:2,560. The gray zone represents the background level. Results are shown as the averages ± S.D. (n=7-8).

Figure S10

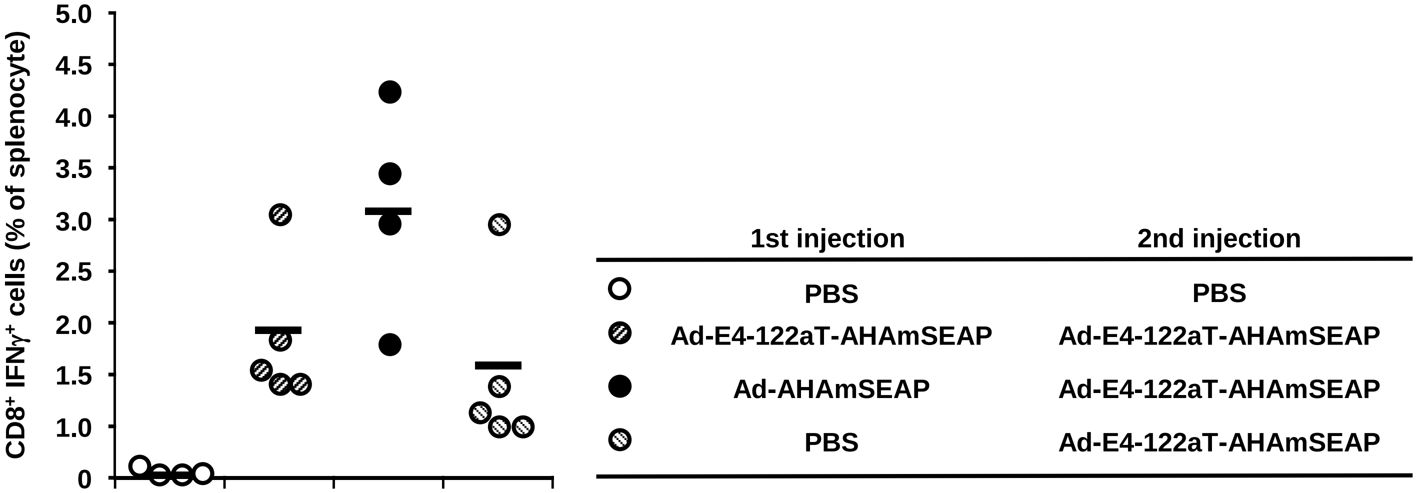

Figure S10. Hexon-specific IFNγ$^+$ CD8$^+$ T cells in the splenocytes following sequential administration of Ad vectors. Neonatal mice were administered Ad vectors at a dose of 5.9x10^{11} IFU/kg *via* the retro-orbital sinus on day 0 and *via* the tail vein on day 42. The splenocytes were harvested on day 56 following neonatal injection and incubated with hexon peptide for 6 hours. Percentages of IFNγ$^+$ CD8$^+$ T cells in the splenocytes were detected by FACS. Results are shown as the averages ± S.D. (n=4-5).
